# Cross-cultural differences through subjective cognition: illustration in translatology with the SSTIC-E in the UAE

**DOI:** 10.3389/fpsyg.2024.1125990

**Published:** 2024-03-07

**Authors:** Emmanuel Stip, Fadwa Al Mugaddam, Karim Abdel Aziz, Leena Amiri, Syed Fahad Javaid, Danilo Arnone, Eisa Almheiri, Abdulla Al Helali, Abderrahim Oulhaj, Yauhen Statsenko, Milos R. Ljubisavljevic, Shamil Wanigaratne, Ovidiu Lungu, Dalia Karpauskaite, Viktorija Aksionova, Aravinthan Subbarayan, Ravi Pralhad Bangalore, Adham Mancini-Marie

**Affiliations:** ^1^Department of Psychiatry, Montreal University, Centre Hospitalier Universitaire de l’Université de Montreal, Instititut Universitaire en Santé mentale de Montréal, Montreal, QC, Canada; ^2^Department of Psychiatry, College of Medicine and Health Science, United Arab Emirates University, Al-Ain, United Arab Emirates; ^3^Department of Psychiatry, University of Ottawa, Ottawa, Canada; ^4^Department of Mental Health, The Ottawa Hospital, Ottawa, Canada; ^5^Centre for Affective Disorders, Psychological Medicine, King’s College London, London, United Kingdom; ^6^Khalifa University, Abu Dhabi, United Arab Emirates; ^7^Montreal University, Montreal, QC, Canada; ^8^New York University Abu Dhabi, Abu Dhabi, United Arab Emirates; ^9^University of Bath, Bath, United Kingdom; ^10^Université de Neuchâtel, Neuchâtel, Switzerland

**Keywords:** SSTICS, subjective cognition, cognitive complaints, memory assessment, meta-cognition, schizophrenia, Arabic translation, cognitive scale

## Abstract

The development of appropriate and valid multicultural and multilingual instruments research is necessary due to a growing multicultural and multilingual society in the 21st century. We explored the use of a cognitive scale related to subjective complaints, focusing on the first step: a cross-cultural and semantic validation. This study presents the translation and cross-validation process of the “Subjective Scale to Investigate Cognition in Schizophrenia” (SSTICS) for the United Arab Emirates (UAE) region via different languages used in Dubaï/Abu Dhabi. This scale measures cognitive complaints and has been validated with psychosis and used in 20 clinical trials worldwide. It evaluates areas of the illness related to self-awareness focusing on memory dysfunction and deficits of attention, language, and praxis. We described the method of cross-cultural validation, with back-translation, semantic steps, and societal contexts. The use of the Subjective Scale to Investigate Cognition in Emirates (SSTIC-E) was explored with different samples of UAE Arabic-speaking subjects. First, a pilot sample mean SSTICS total score was 16.5 (SD:16.9); (*p* < 0.001). The SSTIC-E was then administered to 126 patients and 84 healthy control participants. The healthy group has a lower mean score of 22.55 (SD = 12.04) vs. 34.06 (SD = 15.19). The method was extended to nine other languages, namely, Pakistani/Urdu, Hindi, Marathi, Lithuanian, Serbian, German, Romanian, Sinhala, and Russian. The scales are provided in the article. The overall aim of the translation process should be to stay close to the original version of the instrument so that it is meaningful and easily understood by the target language population. However, for construct validity, some items must be adapted at the time of translation to ensure that the questioned cognitive domain is respected. For example, cooking, an executive function, does not have the same occurrence for an Emirati male, or remembering a prime minister’s name, semantic memory, requires an electoral system to appoint the leader of a country. Translation methods and processes present many challenges but applying relevant and creative strategies to reduce errors is essential to achieve semantic validation. This study aims to measure personally experienced knowledge or attitudes; such language effects can be a thorny problem.

## Introduction

The availability of psychological assessment tools in many languages is essential in generalizing research results across different cultural settings or in conducting cross-cultural comparative studies. Yet, translating these tools, especially those relying on the reporting of subjective states, poses several challenges. According to Nida (1993) and Huang et al. (2022), cultural differences constitute significant obstacles for translators and are the source of misconceptions among readers. If translation is seen as a form of intercultural communication and cultural differences cause significant difficulties in translation, then its impact is even more prominent when it comes to translating subjective cognitive content. According to these authors, in addition to lexical, semantic, and grammatical structures, translation becomes a task that serves to faithfully transcribe the meaning of information written in one language (for instance English) with the same information in another language (Arabic).

Most psychometrically sound research instruments have been developed and their properties evaluated using English-speaking populations (Capitulo et al., 2001; Duffy, 2006). Given the cultural diversity and the variety of languages spoken in the world, health researchers are challenged to be culturally and linguistically sensitive when administering a clinical instrument (Abdel Aziz et al., 2021).

The process of cross-cultural validation allows the translation of a clinical instrument into a language different from the one in which it was developed and it may contribute to identifying any possible cultural-related variability in the phenomena studied when a different language is used. It is always considered a complex process because of the possible interference arising from cross-cultural and ethnic factors that were not accounted for at the time the instrument was originally designed (Duffy, 2006).

Success in tackling these challenges, according to Hilton and Skrutkowski (2002), depends on understanding the fundamental problems of language equivalence, cultural constructs, and psychometric changes that are embedded in translation (Stip, 1996). This should be the first consideration when seeking to develop a culturally equivalent translated instrument. We illustrate this process in psychopathology with the case of a scale measuring cognitive complaints in schizophrenia (Stip, 1996; Hilton and Skrutkowski, 2002).

## Subjective Scale to Investigate Cognition in Schizophrenia

Schizophrenia is associated with significant cognitive impairments, as illustrated by both narrative (Stip, 1996) and systematic reviews, such as a recent umbrella review (Gebreegziabhere et al., 2022) that summarized the findings of 63 systematic reviews published in this field (Table 1).

**Table 1:** Description of the SSTICS items.

Cognition	Evaluating questions	اإلدراك
Memory: Working memory Explicit long-term memory: Episodic memory Semantic memory	Memory: Questions 1, 2 Questions 3–11 Questions 3–9 Questions 10, 11	الذاكرة: • الذاكرة العاملة • الذاكرة الصريحة طويلة المدى: o الذاكرة العرضية o الذاكرة الداللية
Attention: Distractibility Alertness Selective attention Divided attention Sustained attention	Attention: Question 12 Question 13 Question 14 Question 15 Question 16	االنتباه: • الت شتت • اليقظة • االهتمام اإلنتقائي • االهتمام المنقسم • االهتمام المتواصل
Executive functions: Planning Organization Flexibility	Executive functions: Question 17 Question 18 Question 19	الوظائف تنفيذية: • التخطيط • التنظيم • المرونة
Language	Language Question 20	اللغة
Praxia	Praxia Question 21	العمل

Specifically, these systematic reviews have reported that people with schizophrenia have lower cognitive functioning when compared to both healthy individuals and those with affective disorders, with some cognitive domains being affected more than others. The specific deficits relate to long-term outcomes in the areas of memory, attention, executive tasks, language, and social cognition, and most chronic patients with schizophrenia are ranked in the fifth percentile below normal in some measures of neuropsychological function (Tyson et al., 2008).

Generally, the memory includes a set of autonomous systems: sensory memory, short-term memory (working memory), and long-term memory. The latter is divided into explicit (conscious), also known as declarative memory (facts and events), and implicit (unconscious) memory, also known as procedural memory (skills and tasks). Finally, declarative memory is divided into semantic and episodic memory (Craik and Lockhart, 1972).

Among the cognitive impairments reported in schizophrenia, those related to self-perceived insight continue to be a developing research area and one of the utmost clinical importance given the findings that more than half of all patients suffering from schizophrenia do not believe that they have a disorder (Dam, 2006). Moreover, in addition to general cognitive ability and cognitive dysfunctions, poor clinical insight in schizophrenia has been found to also be associated with theory of mind deficits (Bora, 2017), thus highlighting the difficulty of a patient with positive or negative symptoms to subjectively recognize their own cognitive impairment (Lecardeur et al., 2009). Research in the area of insight and cognitive functioning in schizophrenia depends on the availability of assessment tools that, in addition to objective neuropsychological testing, make use of patients’ self-reporting. One such tool is the Subjective Scale to Investigate Cognition in Schizophrenia (SSTICS) developed by Stip et al. (2003). The scale is a questionnaire based on 21 very pragmatic questions that assess specific aspects of cognition and target cognitive domains known to be impaired in people with schizophrenia. This scale was originally constructed and validated in parallel in the two official Canadian languages, French and English. Since its inception, there have been more than 20 publications in PubMed that employed this scale, and it has been used in clinical studies translated into five languages, namely Italian (Stratta et al., 2020), Castilian (Bengochea Seco et al., 2010), Mandarin/Taiwan (Chuang et al., 2019), Korean (Shin et al., 2016), Hindi (Baliga et al., 2020), and Tunisian Arabic or SASCCS (Johnson et al., 2009; Bouhamed et al., 2021; Haddad et al., 2021).

SSTICS is an instrument designed to collect self-reported cognitive symptoms in patients with schizophrenia (Prouteau et al., 2004; Homayoun et al., 2011; Cella et al., 2020). The scale has been constructed and described in a seminal article in 2003 (see Stip et al., 2003 for the complete description of each item of the scale) (Stip et al., 2003). It is straightforward to use and designed as a Likert-type. It provides a measure of all the aspects of subjective cognition and insight into schizophrenia (Bayard et al., 2009; Sellwood et al., 2013; Potvin et al., 2014; Stip et al., 2022).

Initially based on the discoveries of objective cognitive deficits in schizophrenia, the SSTICS scale is a construct that, using simple questions asked to the person, aims to reflect a cognitive complaint in a mirror of the objective cognitive domain. Therefore, specific areas of cognition covered are working memory, explicit memory (divided into episodic and semantic memory), attention, executive function, and language. The scale consists of five domains to assess subjective complaints, in which the first domain assesses memory in two forms (working memory in questions 1 and 2) and explicit long-term memory (episodic memory questions 3–9, and semantic memory in questions 10 and 11). The second domain assesses attention in five sub-domains from question 12 to question 16 (distractibility Q12, alertness Q13, selective attention Q14, divided attention Q15, and sustained attention Q16). The third domain evaluates executive functions in questions 17, 18, and 19, by asking about planning in Q17, organization in Q18, and flexibility in Q19. The fourth domain for language assessment in question 20 and question 21, which is the last on the scale is for praxia assessment (Table 2).

**Table 2:** SSTICS – (Urdu version).

**موضوعی پیمانہ براۓ تحقیق شعور و ادراک بہ مرض شیزوفرینیا**				
اس صفحے پر آپ کچھ فقرہ جات کی فہرست دیکھ رہے ہیں جو کہ آپ کی روزمرہ زندگی میں یاد داشت اور توجہ سے متعلق ہیں۔ آپکو اپنی حالیہ زندگی میں ان مشکلات کے بارے میں اپنی راۓ بیان کرنی ہے۔ ان سب سوالوں کے جواب دیجۓ۔ آپ ان اعداد کو استعمال کر کے اپنے احساسات بیان کر سکتے ہيں**ھدایات:**				
**بار بار-4**	**اکثر-3**	**کبھی کبھی-2**	**شاذ و نادر-1**	**کبھی نہیں-0**
ی آپ نے اپنایک ھے؟ی محسوس کی کوئ کمںی مادداشتی				1
ڈاکٹر کا نام؟ای ہے؟ مثال کے طور پر فون نمبر، کوئ پتہ، کمرہ نمبر، بس کا نمبر ی مشکل ہوتںی استعمال کرنے می رکھنے اور فورادی نئ معلومات کو ی آپ کو کسایک				2
ںیزی آپ کو کچھ چایک فہرست؟ی ناموں کای فہرست ی کانےی ہے؟ مثال کے طور پر کری آتشی مشکل پںی رکھنے مادی				3
ہے؟ی آتشی مشکل پںی رکھنے مادی فہرست ی کاتی ادوی آپ کو اپنایک				4
ں؟ی ڈاکٹر سے طے شدہ ملاقات بھول جاتے ہای اپنے دوستوں سے ملاقات ی آپ کبھایک				5
ں؟ی بھول جاتے ہنای لاتی ادوی آپ اپنایک				6
ںی پڑھتے ہںی ہے جو آپ اخبار می آتشی مشکل پںی رکھنے مادی آپکو اس معلومات کو ایک ں؟ی ہکھتےی پر دوژنیلی ٹای				7
ی آپ کبھای ہے؟ مثال کے طور پر کی آتشی مشکل پںی مرمت کے کاموں مای کام کاج لوی آپکو گھرایک ں؟ی شامل ہںی مبی ترکی کون کون سے اجزاء کھانے کای بنانا ہے سےی کہ کھانا کںی بھول جاتے ہہی				8
نکی آپکو ہسپتال، کلایک ہے؟ی ہوتی دشوارںی رکھنے مادی پھر اپنے گھر کا راستہ ای				9
کا نام؟راعظمی ہے؟ مثلا" پاکستان کے وزی ہوتی دشوارںی رکھنے مادی کے نام اتی آپکو مشہور شخصایک				10
ہے؟ی آتشی مشکل پںی رکھنے مادی کو افتوںی دری اہم سائنسای واقعات، ممالک، براعظم یخی آپکو مختلف ممالک کے دارالخلافے، اہم تارایک				11
ںی ہٹھتےی کا تسلسل کھو بالاتی مثال کے طور پر گفتگو کے دوران توجہ ہٹنے پر آپ اپنے خں؟ی محسوس کرتے ہای کھوای کھوای آپ خود کو غائب دماغ ایک ں؟ی مشکل محسوس کرتے ہںی توجہ برقرار رکھنے می پھر مطالعہ کرتے وقت اپنای				12
ںی آپکو چوکس رہنے مایک ںی ہے؟ مثلا" جب آپ آگ کا الارم سنتے ہی مشکل ہوتںی حالات کا سامنا کرنے مرمتوقعی غای ہے؟ی سے آپ کے پاس سے گزر جاتیزی تی گاڑی جب سڑک پار کرتے ہوئے کوئای				13
وں؟ توجہ بانٹ رہے ہی بات کر کے آپ کںی کے بارے میقی رکھنا جب کچھ لوگ آپ کے سامنے موسادی ڈاکٹر سے طے شدہ ملاقات کو ای دوا کا نام ی مثال کے طور پر اپنں؟ی مشکل محسوس کرتے ہںی کرنے مصلہی فںی آپ اہم معلومات کے بارے مای تو کںی ہی جاتی ساتھ دکیجب آپکو مختلف معلومات ا				14
سمجھا رہا ہو؟ںی دوا کے بارے می گننا جبکہ وہ آپکو آپکسےی دکان پر پی دوا ساز کای کرنا ادی بناتے وقت کوئ پتہ ی مثلا" کافں؟ی وقت دو کام کر سکتے ہکی آپ بایک				15
ۓ کتاب کا مطالعہ کرتے ہوں،ی کانفرنس می مثال کے طور پر کسں؟ی توجہ مرکوز رکھ سکتے ہادہی شے پر 20 منٹ سے زکی ای آپ کسایک ؟ۓ پڑھتے ہوںی جماعت مۂ کمرای				16
؟ی منطوبہ بندی کپڑے دھونے کای کرنے اری کے اخراجات کا حساب، کھانا تنےی مہ،ی منصوبہ بندی ہے؟ مثال کے طور پر سفر کی مشکل محسوس ہوتںی می منصوبہ بندی کاتی مصروفی نسبت اپنی آپکو پہلے کایک				17
کرنا؟ام مرمت کے کلوی کام کاج کرنا، کپڑے دھونا، ذرائع آمد و رفت استعمال کرنا اور گھرلوی کرنا، باہر کے کام کرنا، کھانا پکانا، گھریداری فون استعمال کرنا، کچھ خریلی مثال کے طور پر ٹں؟ی سے انجام دے پاتے ہقےی نسبت اپنے روزمرہ کے کاموں کو مربوط اور منظم طری آپ پہلے کایک				18
؟ۓ آشی پی دشوارںی بدولت کام می کار مختلف ہو جانے کۂقی ہوں پر طری راضی اور آپ بھۓ ہے؟ مثال کے طور پر جب آپکو کچھ مختلف کرنے کو کہا جای آتشی مشکل پںی کو بدلنے مقےی حرکات اور کام کرنے کے طرصلوں،ی آپکو اپنے فایک				19
ہے؟ی آتشی مشکل پںی رکھنے مادی کے نام زوںی چای سمجھنے، تلّفظ ی الفاظ کو استعمال کرنے، فقرے بنانے، الفاظ کے معنحی آپکو صحایک				20
ں؟ی سے تالا کھولنے می پھر چابای اور کانٹے کے استعمال ینچی زپ استعمال کرنے، اوزار کے استعمال، قی ہے؟مثال کے طور پر بٹن بند کرنے، کپڑوں کی مشکل محسوس ہوتںی کھانا کھانے مای آپکو کپڑے بدلنے ایک				21

## UAE and cross-cultural validation context

Mental health services started in the mid-1970s in the UAE and developed from the psychiatric services offered in some of the emirates (i.e., Abu Dhabi and Dubai). In the early 1980s, psychotherapy that was offered to psychiatric patients as treatment in hospitals was seen as a supportive service (Al-Darmaki, 2003, 2004). Over the last 30 years, the need for psychotherapy in the UAE community gained importance with the rapid social and economic changes associated with the influence and impact of other cultures (Table 3).

**Table 3:** SSTICS – (Hindi version).

दी हुई पर्ची पर कु छ वाक्य है जो दै निक कार्यों में र्याादाश्त और ध्यान से सम्बंनित मुश्किलें व्यक्त करते है | आपके व्यव्हार हाल ही में ऐसी घन ाओ का न ुमान लगाकर सभी प्रश्नो का उत्तर दीिजर्ये | उत्तर देन े के िलए िदए गए निकल् ो ं में से सही क्रमांक पर गोला न ार्ये:					
४ - बार बार ३ - अक्सर २ - कभी कभी १ - बहुत कम ० - कभी नहीी ीीं					
1. क्या आपको र्चीज़े र्यााद रश्क े में कोई कनिन ाई महसूस होती है?	४	३	२	१	०
2. क्या आपको ताजी जान कारी र्यााद कररे और उसका तुरं त उपर्योग कररे में कनिन ाई होती है – जैसे िक फ़ोन न ं र, पता, रूम न ं र, स मागग, र्याा डॉक्टर का न ाम?	४	३	२	१	०
3. क्या आपको र्चीज़े स्मरण कररे में कनिन ाई होती है - जैसे िकराणे र्याा न ामों की सूर्ची?	४	३	२	१	०
4. क्या आपको नपे नदाइर्यों के न ाम र्यााद कररे में कनिन ाई होती है?	४	३	२	१	०
5. क्या आप कभी र्चीज़े भूल जाते हैं - जैसे िक दोस्त से िमनला र्याा डॉक्टर से िमनले की ितनि?	४	३	२	१	०
6. क्या आप नपी नदा लेन ा भूल जाते हैं?	४	३	२	१	०
7. क्या आपको ख़ ार में पढ़ी र्याा ीन ी पर सुन ी हुई र र्यााद रश्क े में कनिन ाई होती है?	४	३	२	१	०
8. क्या आपको घर के काम र्याा ररपेर्यार में मुश्किल होती है? जैसे िक ान ा न ान े की निनि र्याा सामग्री?	४	३	२	१	०
9. क्या आपको स्पताल र्याा ाह्य रोग निभाग र्याा ुदके घर का रास्ता र्यााद रश्क े में कनिन ाई होती है?	४	३	२	१	०
10. क्या आपको मशहूर लोगों के न ाम र्यााद कररे में कनिन ाई होती है? जैसे िक भारत के नप्रान मंत्री?	४	३	२	१	०
11. क्या आपको देश की रानजान ी, महत्वपूणग ऐितहािसक िनद, दू सरे देशों के न ाम, र्याा महत्वपूणग न ै ज्ञानिक ोज र्यााद कररे में कनिन ाई होती है?	४	३	२	१	०
12. क्या आप ोए ोए रहते हो? उदाहरण के िलए - ात करते समर्या नपी निर्चााररारा भूल गए, क्योंिक आप निर्चािलत हो गए, र्याा आपको पढ़ते समर्या ध्यान एकाग्र कररे में कनिन ाई होती है?	४	३	२	१	०
13. क्या आपको र्चौकन्ना रनहे में र्याा आपत्कालीन पररिनिितर्यो में प्रितिक्रर्याा कररे में कनिन ाई होती है? जैसे की फार्यार लामग नजे पर र्याा रास्ता पार करते समर्या िकसी गाडी के र्चाान क सानमे नआ े पर?	४	३	२	१	०
14. क्या आपको निनिन छो ी - छो ी जान काररर्यााा ााँएक सान िमनले पर कौन सा महत्वपूणग है, र्ये समन े में कनिन ाई होती है? जैसे िक ज आस पास दो लोग संगीत के निषर्या में ात कर रहे हैं, त आप नपे नदाई के न ाम र्याा डॉक्टर की गली ितनि को दोहरा पाते हो?	४	३	२	१	०
15. क्या आप दो काम एक सान न हीं कर पाते है? जैसे की कॉफ़ी न ाते समर्या पता र्यााद कररा र्याा पैसे िनगन ा ज मेिडकल न ाला आपको आपकी नदा सम ा रहा हो?	४	३	२	१	०
16. क्या आपको एक ही र्चीज़ पर २० िनम से निक ध्यान एकाग्र लररे में कनिन ाई होती है? जैसे िकसी सम्मलेन में र्याा िकता पढ़ते समर्या र्याा कक्षा में पान पढ़ते समर्या?	४	३	२	१	०
17. क्या आपको पहले की तरह आसान ी से कार्याग की र्योनजा न ान ा कनिन लगता है? जैसे की िकसी र्याात्रा पे जान े की र्योनजा, मािसक र्चाग, ान ा न ान ा र्याा कपडे न ोन े के िलए समर्या निकानला आिद?	४	३	२	१	०
18. क्या आपको पहले जो दै निक िक्रर्यााएाााँ आसान ी से कर पाते न े, न ह कररा कनिन लगता है? जैसे की फ़ोन का इस्तेमाल, दुकान से सानम लान ा, ान ा न ान ा, र्याातार्याात का उपर्योग, आिद?	४	३	२	१	०
19. क्या आपको र्यािद र्चााल, निणगर्या र्याा काम कररे का तरीका दनले को कहा जार्या और आप	४	३	२	१	०
20. क्या आपको शब्द इस्तेमाल कररे में, न ाक्य न ान े में, शब्दों का न ग समन े में, उच्चारण में र्याा र्चीज़ों के न ाम र्यााद कररे में कनिन ाई होती है?	४	३	२	१	०
21. क्या आपको कपडे पनहन े में र्याा ान े में कनिन ाई होती है? जैसे न लगान ा, कैं र्ची प्रर्योग कररा, र्चामर्चा का इस्तेमाल, ताला लगान ा आिद?	४	३	२	१	०

Only recently, several objective assessment tools have been translated and adapted for application in the UAE Mini-Mental State Examination (MMSE) and Montreal Cognitive Assessment test (MOCA). However, there are no assessment tools to assess subjective cognitive complaints or deficits in performance in patients with schizophrenia in the UAE.

The presence of interpreters or translators may complicate the psychotherapeutic process of a patient (El-Islam, 2005; Miller et al., 2005; Anderson et al., 2009; Hunt and Swartz, 2017). A clear understanding of the dialectal and cultural characteristics among Arab patients is needed (Okasha et al., 2000; Sayed, 2003). The development of mental health concepts depends on the accuracy of the conversation between the therapist and the patient, as well as the impact of culture on how the patients can express their emotions. Personal emotional expression, either publicly or in interactions with others, is not encouraged in UAE culture in general (Table 4).

**Table 4:** SSTICS – (Marathi version).

सूर्चिा: तुम्हाला नदलेल्या कानगावर न ै िंनदि कामकाजात स्मरणशक्ती आदण एकाग्रता र्या ा तीत होणार्याा डर्चणी द्दल मादहती र्यान ी नदलेली आहे. तुमच्या व्यवहारात होणार्याा घ िांर्चे मोजमाप करूि ाली नदलेल्या प्रश् ा ं र्ची उत्तरे द्या. उत्तर न े ण्यासाठी तुम्हाला र्योग्य वा ेल तो पर्याार्य दिवडू ि क्रमांकावर गोल दर्चन्ह करा.					
४ - वारीीं वार ३ - अनेकदा २ - कधी कधी १ - क्वचित ० - कधीच नाही					
1. तुम्हाला गोष्टी लक्षात ठे वण्यात डर्चण जाणवली आहे का?	४	३	२	१	०
2. तुम्हाला िुकतीर्च दमळालेली मादहती लक्षात ठे वणे आदण ती पुन्हा लगेर्च वापरणे कठीण जाते का? नउा: फोिे िं र, पत्ता, रूम िं र, स मागा, दकं वा डॉक्टरर्चे िाव.	४	३	२	१	०
3. दकराणा सामाि दकं वा िावांर्ची र्यान ी लक्षात ठे वणे कठीण जाते का?	४	३	२	१	०
4. तुम्हाला तुमच्या औषधांर्ची िावे लक्षात ठे वणे कठीण जाते का?	४	३	२	१	०
5. दमत्राला भे णे दकं वा डॉक्टरला भे ण्यार्ची तारी शा गोष्टी तुम्ही कधी दवसरता का?	४	३	२	१	०
6. तुम्ही तुमर्ची औषधे घ्यार्यला दवसरता का?	४	३	२	१	०
7. तुम्हाला वतामाि पत्रात वार्चलेली दकं वा न ू ररशाि वर ऐकलेली मादहती लक्षात ठे वार्यला कठीण वा ते का?	४	३	२	१	०
8. तुम्हाला घरर्ची कामे दकं वा न ु रुस्ती करतांिा डर्चण होते का? नउा: स्वर्यंपाक करार्यर्ची पद्धत दकं वा त्या साठी वापरले जाणारे सादहत्य दवसरता का?	४	३	२	१	०
9. तुम्हाला न वा ान्यात जार्यर्चा दकं वा नगी स्वतःच्या घरी जार्यर्चा रस्ता ही दवसरार्यला होतो का?	४	३	२	१	०
10. तुम्हाला सुप्रदसद्ध व्यक्तीर्चं ी िावे लक्षात ठे वणे कठीण जाते का? नउा: भारतार्चे प्रधाि मंत्री?	४	३	२	१	०
11. तुम्हाला न े शाच्या राजधान्या, महत्वपूणा ऐदतहादसक घ िा, महत्वपूणा वैज्ञादिक शोध दकं वा न ु सर्याा न े शांर्ची िावे लक्षात ठे वणे कठीण जाते का?	४	३	२	१	०
12. तुम्ही दवसराळू आहात का? स्वतःच्यार्च दवर्चारात मग्न सता का? नउा: संभाषणात मध्येर्च लक्ष दवर्चदलत ाल्यामुळे ोलण्यार्चा धागार्च दवसरूि जाता का दकं वा वार्चतािा लक्ष कें नदत करण्यास ूप कष्ट घ्यावे लागतात का?	४	३	२	१	०
13. तुम्हाला सतका राहणे दकं वा िपेदक्षत पररश्कथितीमं ध्ये प्रदतदक्रर्या न े णे कठीण जाते का? नउा: आगीर्ची सूर्चिा ल्यावर दकं वा रस्त्यात र्चािक वेगवाि गाडी आपल्या समोरूि गेल्यावर?	४	३	२	१	०
14. एकार्च वेळी दवदवध प्रकारर्ची मादहती समोर आल्यावर त्यातली कोणती गोष्ट महत्वार्ची हे सम णे कठीण जातं का? नउा: आजू ाजूला न ोि जि संगीत दवषर्यी र्चर्चाा करत सतािा तुमच्या औषधार्चे िाव दकं वा डॉक्टरला भे ण्यार्ची पुढर्ची तारी लक्षात ठे वणे.	४	३	२	१	०
15. तुम्हाला न ोि गोष्टी एकार्च वेळी करणे जमत िाही का? नउा: कॉफी िवत सतािा ए ान ा पत्ता स्मरण करणे दकं वा मेदडकल वाला आपली औषधे कशी घ्यार्यर्ची हे समजावत सतािा स्खशातले पैसे मोजणे?	४	३	२	१	०
16. तुम्हाला २० दमदि ांपेक्षा जास्त कोणत्याही गोष्टीवर लक्ष कें नदत करणे कठीण होते का? नउा: र्चर्चाासत्र, वार्चि दकं वा वगाात दशकवत सतािा?	४	३	२	१	०
17. तुम्हाला कामार्ची र्योजिा पदहल्या सार ं सहजतेिे करणे कठीण जाते का? नउा: प्रवासाला न्यार्यच्या सामािार्ची र्यान ी करणे, मादसक र्चा, जेवण िवार्यला?	४	३	२	१	०
18. तुम्हाला न ै दिक दक्रर्या पदहल्या इतक्या सहजतेिे करणे कठीण वा ते का? नउा: फोिर्चा वापर, रे न ी करणे, जेवण िवणे, प्रवास करणे, वस्तू न ु रुस्त करणे इत्यान ी.	४	३	२	१	०
19. तुम्हाला गरज पडली तर गोन र ठरवलेले दिणार्य, काम करण्यार्ची पद्धत प त सतािा न े ील आर्यत्या वेळी न लणे कठीण जाते का?	४	३	२	१	०
20. तुम्हाला श ् ा ं र्चा वापर, वाक्य िवणे, श ् ार्चा न ा समजणे, उच्चार करणे, वस्तूंर्ची िावे आठवणे कठीण जाते का?	४	३	२	१	०
					
21. तुम्हाला कपडे घालणे दकं वा जेवणे र्या गोष्टी कठीण वा तात का? नउा: कपड् ा ं र्ची णे लावणे, कात्री वापरणे, र्चमर्चा वापरणे, कु लूप लावणे.	४	३	२	१	०

Patients are encouraged by their therapists and physicians to express their emotions and describe how their overall health status is affected. The same concerns can be applied to cognition. Therefore, considering the differences between cultures, languages, and beliefs is crucial when developing or modifying a psychometric scale, ensuring the relevance and effectiveness of the resulting instrument.

### Relevance

For any novel psychometric instrument to be applied in a different culture (UAE) from the setting in which it was originally developed (Canada), it needs to be appropriately adapted. Cultural adaptation of an existing instrument has many benefits over creating an entirely new tool, such as reducing cost and time spent developing it. Psychometric instruments, such as SSTICS, can not only stimulate cross-cultural research but also be employed in many fields such as public health, primary health care, psychiatry, neurology, and other disciplines. In practice, cultural adaptation is done while translating the tool from its original language and it precedes its psychometric validation through the assessment of different aspects of reliability and validity using well-established scientific measures. In this process, the self-reporting scales are potentially susceptible to significant alterations resulting from a series of influences, including social attractiveness, suppression, habits, and response style. Thus, describing and analyzing the cultural adaptation and psychometric validation process may serve as a guide for future similar undertakings (Table 5).

**Table 5:** SSTICS – (Lithuanian version).

Subjektyvi skalė tirti pažinimą šizofrenijoje	
Instrukcijos: Priešais save matote frazių sąrašą, apibūdinantį atminties ar susikaupimo problemas, kurias kiekvienas iš jūsų galite pamatyti savo kasdienėje veikloje. Jūsų prašoma įvertinti pastaruoju metu pastebėtų tokių sutrikimų elgesį, atsakant į visus klausimus. Naudokite reitingų skalę, apibraukite artimiausią skaičių, ką jaučiate.	
4- labai dažnai − 3- dažnai − 2- kartais − 1- retai − 0- niekada	
1 - Ar pastebėjote sunkumų prisimindami dalykus?	
2 - Ar jums sunku prisiminti naujai gautą informaciją, kurią būtina naudoti nedelsiant, pvz., telefono numerį, adresą, kambario numerį, autobuso maršruto numerį ar gydytojo vardą?	
3 - Ar jums sunku įsiminti dalykus, pvz., maisto prekių sąrašą ar vardų sąrašą?	
4 - Ar sunku prisiminti savo vaistų pavadinimus?	
5 - Ar kada pamiršote dalykus, tokius kaip pasimatymas su draugu ar susitikimas su gydytoju?	
6- Ar pamirštate išgerti vaistus?	
7 - Ar sunku prisiminti informaciją, kurią skaitėte laikraščiuose ar girdėjote per televizorių?	
8 - Ar jums sunku atlikti namų ruošos darbus ar remontuoti? Pavyzdžiui, ar kada pamiršote, kaip gaminti, ar kokie ingredientai pateikiami recepte?	
9 - Ar sunku prisiminti, kaip patekti į ligoninę, polikliniką ar net į savo namus?	
10 - Ar jums sunku prisiminti žinomų žmonių, tokių kaip Kanados ministras pirmininkas, vardus?	
11 - Ar jums sunku prisiminti nacionalines sostines, svarbias istorijos datas, kitų žemynų šalių pavadinimus ar svarbiausius mokslo atradimus?	
12 - Ar esate nesąmoningas, ar esate debesyse? Pavyzdžiui, prarandate minties kryptį pokalbyje, nes esate išsiblaškęs arba jums sunku susitelkti ties tuo, ką skaitote?	
13 - Ar sunku būti budriam ar reaguoti į netikėtas situacijas? Pavyzdžiui, priešgaisrinė signalizacija ar automobilis, staiga pravažiuojantis jums pereinant gatvę.	
14 - Ar jums sunku išsiaiškinti, kas svarbu, kai jums vienu metu pateikiama daug įvairios informacijos? Pavyzdžiui, jūsų vaisto pavadinimas arba kito gydytojo paskyrimas, kai du žmonės netoliese kalba apie muziką.	
15 - Ar nesugebi daryti dviejų dalykų vienu metu? Pavyzdžiui, atsiminkite adresą ruošdami kavą arba suskaičiuokite pinigus piniginėje, kol vaistininkas jums paaiškins jūsų vaistus.	
16 - Ar kyla problemų sutelkiant dėmesį į tą patį dalyką daugiau nei 20 minučių? Pavyzdžiui, konferencijoje ar skaitant knygą ar pamokos metu klasėje.	
17 - Ar jums sunku planuoti savo veiklą taip lengvai, kaip anksčiau? Pvz., kelionės maršruto, kaip patekti į vietą, sudarymas, mėnesio biudžeto sudarymas, patiekalų paruošimas ar laiko skyrimas skalbimui.	
18 - Ar jums sunku koordinuoti savo kasdienio gyvenimo judesius ir veiksmus taip lengvai, kaip anksčiau? Pavyzdžiui, naudojimasis telefonu, apsipirkimas, reikalų tvarkymas, patiekalų ruošimas, namų ruošos darbai, skalbimas, keliavimas, namų remontas.	
19 - Ar jums sunku pakeisti savo judesius, sprendimus ar dalykų atlikimo būdus, jei jūsų to paprašo ir jūs sutinkate? Pavyzdžiui, jūs sutinkate tai padaryti, bet sunku, nes tai nebe tas pats.	
20 - Ar jums sunku rasti žodžius, formuoti sakinius, suprasti žodžių prasmę, tarti žodžius ar įvardyti daiktus?	
21 - Ar sunku apsirengti ar valgyti? Pavyzdžiui, tvarkymo mygtukai, užtrauktukai, darbo įrankiai, žirklės, šakutė, raktas spynoje.	

### SSTICS in Arabic

Cross-cultural validation of an assessment instrument is a complex process that requires a significant investment in time and financial resources (Bullinger et al., 1993). Even though a 2015 review of cross-cultural adaptation guidelines for questionnaires did not find a consensus among the 31 guidelines investigated in this study, the authors reported that similar results were achieved using most of them as long as the adaptation and validation processes were properly and separately considered (Epstein et al., 2015). Before venturing into this process for SSTICS, it is vital to ensure that there is no equivalent instrument in the Arabic language, or an equivalent instrument translated and validated. Otherwise, the researcher must make an exhaustive review of the instruments relevant to his study to select the one that has passed the validation stages, is the most rigorous, and is in its original language (Haccoun, 1987; Flaherty et al., 1988; Guillemin et al., 1993).

In general, the cross-cultural validation of an instrument involves three main steps: (1) the translation and verification of its equivalence; (2) empirical verification of the validity of the translated version; (3) adaptation of scores to cultural context and development standards (Epstein et al., 2015). Each of them also includes steps necessary for creating a valid version, and several options are available to the researcher with their advantages and disadvantages. The semantic aspect of a questionnaire is the first step to consider before going any further with the validation. It is often a neglected step. The difference between dialects needs to be considered (Table 6).

**Table 6:** SSTIC – (Serbian version).

Subjektivna skala za procenu kognicije u šizofreniji
Uputstvo: Na papiru ispred sebe vidite listu rečenica koje opisuju probleme sa pamćenjem ili koncentracijom koje možete doživeti u svakodnevnim aktivnostima. Od vas se traži da procenite učestalost poremećaja koje u poslednje vreme primećujete (u svakodnevnim aktivnostima), tako što ćete odgovoriti na sva pitanja. Koristite skalu ocenjivanja tako što čete zaokružiti broj koji najbliže odgovara onome što primećujete (osećate).
4- vrlo često − 3- često − 2- ponekad − 1- retko − 0- nikada
1- Da li ste primetili bilo kakve poteškoće u pamćenju?
2- Da li imate poteškoća s pamćenjem novoprimljenih informacija koje morate odmah koristiti, kao što su telefonski broj, adresa, broj sobe, broj autobuske rute ili ime lekara?
3- Da li imate poteškoće sa pamćenjem stvari, poput liste namirnica ili liste imena?
4- Da li imate poteškoća s pamćenjem imena lekova koje uzimate?
5- Da li ikada zaboravite stvari, poput sastanka sa prijateljem ili zakazanog pregleda kod lekara?
6- Da li zaboravljate da uzimate lekove?
7- Da li imate poteškoća s pamćenjem informacija koje ste pročitali u novinama ili čujete na TV?
8- Da li imate poteškoća u obavljanju kućnih poslova ili popravci? Na primer, da li ikada zaboravite kako da skuvate jelo ili koji sastojci ulaze u recept?
9- Da li imate poteškoća da se setite kako da dođete do bolnice ili ambulante ili čak do svoje kuće?
10- Da li imate poteškoća s pamćenjem imena poznatih ljudi, poput predsednika Srbije?
11- Da li imate poteškoća s pamćenjem nacionalnih prestonica, važnih datuma u istoriji, imena država na drugim kontinentima ili velikih naučnih otkrića?
12- Da li ste odsutni ili ste izgubljeni u mislima? Na primer, da li tokom razgovora izgubite tok misli jer ste rasejani ili se teško fokusirate na ono što čitate?
13- Da li imate poteškoće da budete u pripravnosti ili reagujete na neočekivane situacije? Na primer, na protivpožarni alarm ili automobil koji naglo projuri dok prelazite ulicu.
14- Da li imate poteškoća da razaznate šta je važno kad vam se istovremeno prikažu različite informacije? Na primer, naziv leka ili sledeći pregled kod lekara dok dvoje ljudi istovremeno razgovaraju o muzici u blizini.
15- Da li ste u stanju da uradite dve stvari odjednom? Na primer, da zapamtite adresu dok kuvate kafu ili brojte novac u novčaniku dok vam farmaceut objašnjava lekove.
16- Da li imate problema sa fokusiranjem pažnje na istu stvar duže od 20 minuta? Na primer, tokom konferencije ili tokom čitanja knjiga ili tokom lekcije u učionici.
17- Da li imate poteškoća sa planiranjem svojih aktivnosti podjednako lako kao nekada? Na primer, da izradite plan puta kako biste negde stigli, da napravite budžet za sledeći mesec, da pripremite obrok ili odvojite vreme za pranje veša.
18- Da li imate poteškoća s koordinacijom svakodnevnog života i svakodnevnim aktivnostima kao nekada? Na primer, korišćenje telefona, kupovina, obavljanje poslova, pripremanje obroka, kućni poslovi, pranje veša, prevoz, kućne popravke.
19- Da li imate poteškoća u promeni pokreta, odluka, ili načina na koji obavljate stvari ako se to od vas zatraži i vi sa tim zahtevom slažete? Na primer, vi se slažete da to učinite, ali je teško jer to više nije isto.
20- Da li imate poteškoća u pronalaženju reči, formiranju rečenica, razumevanju značenja reči, izgovaranju reči ili imenovanju predmeta?
21- Da li imate poteškoća sa oblačenjem ili jelom? Na primer, u rukovanju sa dugmadima, patentnim zatvaračima, radnim alatima, makazama, viljuškom, ili ključem u bravi.

There are different types of translation methods. The translation process must ensure that an instrument retains inferential equivalence, that is, it is possible to produce the same inferences from the translated version as with the original instrument. The correspondence between words (semantic equivalence) is difficult to achieve from one culture to another, considering the vocabulary, dialect, and grammar specific to each language.

This can be the case between the UAE and Canada. Some translated expressions have no meaning in another culture, and expressions specific to the target culture, and retaining the meaning of the items must be found (equivalence of expression). Certain situations that are evoked in the culture of the original instrument may not correspond to the reality in another culture. These items should be replaced by other situations appropriate to the target culture while preserving the objective and the meaning aimed by these items (experiential equivalence) (Guillemin et al., 1993).

Finally, the same exercise should be applied to some concepts, which, when translated, do not allow the exact representation of the target culture (conceptual equivalence). Translating items into different contexts is a delicate task that raises many questions. Only translating an instrument into the local language does not guarantee validity and accuracy in a different context.

Capitulo et al. (2001) identified the decentering of the translation process as an extension of back-translation (Capitulo et al., 2001). Once the final version has been developed, after the back-translation, whoever owns the copyright to the original instrument and the translation team/individual will agree on the semantic equivalence between the “original’ and the translation instrument by modifying the original instrument for universally better-understood language (Capitulo et al., 2001).

Hilton and Skrutkowski (2002) describe symmetric and asymmetric categories of translation. Symmetric translation requires that the original and the translation instruments be faithful to meaning and familiarity. Decentering refers to the translation process in which the source and target of language versions are seen as equally important and subject to change (Hilton and Skrutkowski, 2002).

In the asymmetric translation category, the original language is kept without modification, thus the translated version may present a literal translation of words and may lack conceptual equivalence. One of the reasons that an instrument developer or organization resists making changes is to avoid compromising the validity and instrument reliability. The translation process is considered incomplete until the instrument is pretested with members of the target culture.

Pretesting an instrument primarily serves two purposes: it checks the quality of the translation and the practicalities of test administration (Hilton and Skrutkowski, 2002).

Several objectives are targeted when translating the tool, not only the meaning but also the equivalence of content, technique, and concept. The ‘subject’s belonging to a different ethnic group influences their results in the various clinical tests’. A great deal of documentation exists revealing the difficulties caused by using assessment instruments in a different cultural context other than the one they were created for. Flaherty et al. (1988) show that to ensure the correct use of a diagnostic instrument in a new cultural context (Flaherty et al., 1988), it is essential to verify the equivalence of these five dimensions:

Content equivalence corresponds to the content of each instrument item that is relevant in the cultures where its use is conceived. This step includes the verification of the items by a team of specialists from each culture. They decide on the relevance or not of each item. At best, the author of the tool is contacted.

Semantic equivalence corresponds to the fact that the meaning of each item remains the same after translating the tool into the target language. Back-translation is considered the best method to ensure semantic equivalence, translating the scale into the desired language and then reverse translation by another translator into the original language.

A high-importance concept, technical equivalence should be directly related to the assessment method and not interfere with results obtained from one culture to another, for example, when the paper-and-pencil method is not necessarily familiar or a male interviewer interviewing women in certain contexts, which can skew the data.

- Criteria equivalence is the tool’s ability to assess a variable in two different cultures. After analyzing the data, the interpretation of the results should be similar.
- Finally, conceptual equivalence corresponds to the fact that the tool measures the same theoretical construct in each culture, in our case, subjective cognition (Flaherty et al., 1988).

Translation is the first step; the adaptation process is the second step. The term “adaptation” has a different meaning from “translation”; hence, adaptation contains all modifications of the translated document related to cultural, dialectal, linguistic, and contextual aspects (Beaton, 2010; Gjersing, 2010; Paula, 2014). Adaptation ensures that the resulting tool achieves the required uniformity and offers contextual, dialectal, experiential, and hypothetical equivalence, as backed by the expert committee review and the creators of the scale (Bravo et al., 1991).

### Objectives

In this study, our general objective was to present the different steps necessary at the very beginning of the process of cultural adaptation and validation of the SSTICS. In the UAE, the questionnaire’s clientele is from several countries and with various ethnicities making the study complex. We started the back-translations with the Emirates Arabic language, which is the predominant language in the United Arab Emirates, and then we added a comparison of back-translations from 9 other languages for the scale to compare the differences between all back-translations. All the versions will be added to the first Arabic back-translation since UAE has residents from different countries and nationalities (Table 7).

**Table 7:** SSTIC – (German version).

Subjektive Skala zur Untersuchung der Wahrnehmung (SSZUW)
Anweisungen: Auf dem Blatt vor Ihnen sehen Sie eine Liste von Aussagen, die Probleme mit dem Gedächtnis oder der Konzentration beschreiben und die Sie vielleicht bei Ihren täglichen Handlungen erleben. Sie sollen beurteilen, wie häufig Sie diese Störungen in letzter Zeit in Ihrem Verhalten beobachtet haben, indem Sie alle Fragen beantworten. Nutzen Sie die Bewertungsskala und kreisen Sie die Zahl ein, die Ihrem Empfinden am nächsten kommt.
4 – sehr oft, 3 – oft, 2 – manchmal, 1 – selten, 0 – nie
1. Haben Sie Schwierigkeiten, sich an Dinge zu erinnern?
2. Haben Sie Schwierigkeiten, sich an Informationen zu erinnern, die Sie gerade erhalten haben und die Sie sofort wieder nutzen müssen, wie z. B. eine Telefonnummer, eine Adresse, eine Zimmernummer, die Nummer einer Buslinie oder den Namen eines Arztes?
3. Haben Sie Schwierigkeiten, sich Dinge einzuprägen, zum Beispiel die Einkaufsliste oder eine Liste mit Namen?
4. Haben Sie Schwierigkeiten, sich an die Namen Ihrer Medikamente zu erinnern?
5. Vergessen Sie Dinge, zum Beispiel eine Verabredung mit einem Freund oder einen Arzttermin?
6. Vergessen Sie, Ihre Medikamente einzunehmen?
7. Haben Sie Schwierigkeiten, sich an Informationen zu erinnern, die Sie in der Zeitung gelesen oder im Fernsehen gehört haben?
8. Haben Sie Schwierigkeiten, Haushaltsarbeiten oder Reparaturen durchzuführen? Vergessen Sie zum Beispiel, wie bestimmte Sachen gekocht werden oder welche Zutaten zu einem bestimmten Rezept gehören?
9. Haben Sie Schwierigkeiten, sich daran zu erinnern, wie Sie zum Krankenhaus oder zur Ambulanz oder nach Hause kommen?
10. Haben Sie Schwierigkeiten, sich an die Namen bekannter Personen zu erinnern, wie z. B. den Namen eines Staatsführers?
11. Haben Sie Schwierigkeiten, sich an Landeshauptstädte, wichtige Daten in der Geschichte, Namen von Ländern auf anderen Kontinenten oder wichtige wissenschaftliche Entdeckungen zu erinnern?
12. Sind Sie unaufmerksam oder geistesabwesend? Verlieren Sie zum Beispiel den Faden in einem Gespräch, weil Sie abgelenkt sind, oder fällt es Ihnen schwer, sich darauf zu konzentrieren, was Sie lesen?
13. Haben Sie Schwierigkeiten, wachsam zu sein oder auf unerwartete Situationen zu reagieren? Zum Beispiel auf einen Feueralarm oder ein Auto, das plötzlich an Ihnen vorbeirast, wenn Sie die Straße überqueren.
14. Haben Sie Schwierigkeiten, zu entscheiden, welche Information wichtig ist, wenn Sie verschiedene Informationen gleichzeitig erhalten? Zum Beispiel wenn Ihnen der Name Ihres Medikaments oder Ihr nächster Arzttermin mitgeteilt wird, während sich zwei Personen in der Nähe über Musik unterhalten.
15. Sind Sie nicht in der Lage, zwei Dinge auf einmal zu tun? Zum Beispiel sich eine Adresse einzuprägen, während Sie Kaffee zubereiten oder das Geld in Ihrer Geldbörse zu zählen, während der Apotheker Ihnen die Einnahme Ihres Medikaments erklärt.
16. Fällt es Ihnen schwer, Ihre Aufmerksamkeit länger als 20 Minuten auf eine Sache zu konzentrieren? Zum Beispiel bei einer Konferenz oder einer Buchlesung oder während einer Unterrichtsstunde im Klassenzimmer.
17. Fällt Ihnen das Planen Ihrer Aktivitäten nicht mehr so leicht wie früher? Zum Beispiel eine Route zu planen, um irgendwohin zu gelangen, ein Budget für den Monat festzulegen, Mahlzeiten zuzubereiten oder sich Zeit für das Wäschewaschen zu nehmen.
18. Fällt Ihnen die Koordinierung Ihrer Bewegungen und Handlungen im Alltag nicht mehr so leicht wie früher? Zum Beispiel die Nutzung des Telefons, einkaufen gehen, Besorgungen machen, Mahlzeiten zubereiten, Haushaltsarbeiten erledigen, Wäsche waschen, Transportmittel nutzen, Reparaturen zu Hause vornehmen.
19. Haben Sie Schwierigkeiten, Ihre Bewegungen, Entscheidungen oder die Art und Weise wie Sie gewisse Dinge tun, zu ändern, wenn Sie dazu aufgefordert werden und damit einverstanden sind? Zum Beispiel stimmen Sie zu, etwas zu tun, aber es ist schwer, weil es nicht mehr dasselbe ist.
20. Fallen Ihnen bestimmte Wörter nicht ein, haben Sie Schwierigkeiten, Sätze zu formulieren, die Bedeutung von Wörtern zu verstehen, Wörter auszusprechen oder Gegenstände zu benennen?
21. Haben Sie Schwierigkeiten beim Anziehen oder Essen? Zum Beispiel beim Umgang mit Knöpfen, Reißverschlüssen, Werkzeugen, Scheren, mit der Gabel, einem Schlüssel im Schloss.

Specifically, we aimed to (1) translate the SSTICS into UAE Arabic; (2) explain the cultural adjustment needed to make the scale applicable and adjusted to suit the UAE; (3) present and post the scale and make it available to clinicians, readers, and researchers who are planning to validate it in the UAE. (4) We tested the feasibility of it in the UAE with a pilot (*N* = 13) and an exploratory study (*N* = 23). (5) Additionally, we added comparisons for the scale back-translations in different 9 languages, that can be used and validated in those countries, as well as for patients with those nationalities living in UAE such as Egyptian Arabic, Pakistan/Urdu, Hindi, Marathi, Lithuanian, Serbian, German, Romanian, Sinhala, and Russian (Table 8).

**Table 8:** SSIC – (Romanian version).

Scala Subiectivă pentru Investigarea Cogniției
Instrucțiuni: Pe această foaie veți vedea o listă de fraze care descriu probleme ale memoriei sau legate de concentrare pe care fiecare dintre dumneavoastră le puteți întâlni în viața de zi cu zi. Sunteți rugați să estimați frecvența acestor probleme pe care le-ați observat recent în comportamentul dumneavoastră, răspunzând la toate întrebările. Utilizați scala de evaluare încercuind numărul care corespunde cel mai aproape de ceea ce simțiți.
4- foarte des; 3-adesea; 2-câteodată; 1-rar; 0- niciodată
1- Ați observat vreo dificultate amintindu-vă lucruri?
2- Aveți difficultăți reținând informații pe care le-ați primit de curând și care trebuie utilizate imediat, *cum* ar fi un număr de telefon, o adresă, un număr de cameră, un număr de autobuz sau numele unui doctor?
3- Aveți difficultăți memorând lucruri precum o listă de cumpărături sau o listă cu nume?
4- Aveți difficultăți amintindu-vă numele medicamentelor dumneavoastră?
5- Uitați vreodată lucruri precum o intâlnire cu un prieten sau o vizită la doctor?
6- Uitați vreodată să vă luați medicamentele?
7- Aveți difficultăți amintindu-vă informații pe care le-ați citit în ziare sau le-ați auzit la TV?
8- Aveți difficultăți făcând treburile casei sau reparații? Spre exemplu, ați uitat vreodată *cum* sa gătiți ceva sau ce ingrediente trebuie la o rețetă? Do you have difficulty doing household chores or repairs?
9- Aveți difficultăți amintindu-vă *cum* să ajungeți la spital sau la policlinică sau chiar la domiciliul dumneavoastră?
10- Aveți difficultăți amintindu-vă numele oamenilor faimoși, *cum* ar fi președintele țării?
11- Aveți difficultăți amintindu-vă capitale naționale, date importante din istorie, numele țărilor de pe alte continente sau descoperiri științifice majore?
12-. Sunteți pierdut sau cu mintea in nori? Spre exemplu, vă pierdeți șirul gândurilor într-o conversație din cauză ca sunteți distrat sau aveți difficultăți concentrându-vă la ceea ce citiți?
13- Aveți difficultăți să rămâneți alert sau să reacționați la situații neașteptate? Spre exemplu, la o alarmă de incendiu ori o mașină care trece brusc pe lângă dumneavoastră când treceți strada?
14- Aveți difficultăți să identificați ceea ce este important atunci când vă sunt prezentate mai multe informații simultan? Spre exemplu, numele medicamentelor dumneavoastră sau următoarea vizită la doctor in același timp în care doi oameni vorbesc despre muzică în apropiere.
15- Sunteți incapabil să faceți două lucruri în același timp? Spre exemplu, să memorizați o adresă în timp ce faceți cafea sau să numărați banii din portofel în timp ce farmacistul vă explică *cum* să luați medicamentele.
16- Aveți vreo problemă concentrându-vă atenția asupra aceluiași lucru pentru mai mult de 20 de minute? Spre exemplu, la o conferință, lectură de carte sau în timpul unei ore in clasă?
17- Aveți difficultăți să vă planificați activitațile așa *cum* obișnuiați să o faceți? Spre exemplu, stabilind itinerariul pentru a merge undeva, făcând bugetul lunii, pregătind de mâncare sau planificându-vă spălatul rufelor.
18- Aveți difficultăți coordonându-vă mișcările și acțiunile în viața zilnică așa *cum* obișnuiați să o faceți? Spre exemplu, folosind telefonul, făcând anumite cumpărături sau comisioane, pregătind de mâncare, făcând treburi prin casă, spălând rufe, folosind mijloacele de transport, făcând reparații în casă.
19- Aveți difficultăți schimbându-vă mișcările, deciziile sau felul in care faceți lucruri atunci când sunteți rugat să o faceți și sunteți de acord cu asta? Spre exemplu, sunteți de acord să o faceți, dar vă vine greu pentru că nu mai este la fel.
20- Aveți difficultăți să vă găsiți cuvintele, să formulați propoziții, să înțelegeți sensul cuvintelor, pronunțarea lor sau numirea obiectelor?
21- Aveți difficultăți să vă îmbrăcați sau sa mâncați? Spre exemplu, cand vine vorba de manipularea nasturilor, fermoarelor, uneltelor de lucru, foarfecelor, furculiței sau cheii în ială?

### Method (Arabic)

The initial translation aims to develop the first version of the tool in the language used in the UAE. A native speaker of both languages Arabic and English did the initial translation. The first step in developing a subjective scale to investigate cognitive deficits in the Emirate population was performed through four main stages. The first stage was the examination of the two versions of (SSTICS), the initial French language version that was translated to Tunisian Arabic (SACCS) and the English language version (SSTICS). The second stage was the translation (by a single researcher) of the English SSTICS into a second translated Arabic version in the Emirates Arabic language, named the Subjective Scale to Investigate Cognition in Emirates (SSTICE).

**Figure 1:**
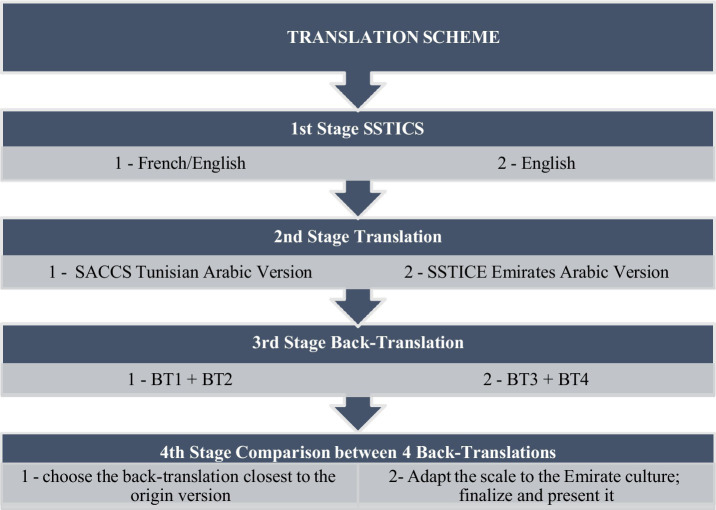
Translation scheme.

The third stage involved translating the two Arabic SSTICS versions (SACCS and SSTICE) back into the English language. Two independent researchers processed each scale version, each blind to the other “investigator’s work.” After completing this stage, we obtained four different back-translations (two back-translations for each scale), performed by four independent researchers. In the fourth stage, one reviewer compared the four translations and chose the version most like the original English-language SSTICS, so that adaptation and validation could be performed, and the final version of SSTICE-GCC was presented (Figure 1). The author of the scale (ES) reviewed all the versions.

During the harmonization stage, meetings were held between the different people involved in translation to examine the divergent items and concepts and ensure inter-translation validity. During these meetings, participants made decisions regarding concepts identified as potentially problematic or confusing in the instructions’ meaning and understanding. When necessary, the author of the tool has provided clarifications on the meaning of the concepts so that the Arabic version is as faithful as possible to the original. Decisions made by meeting participants were noted, and a draft of the tool was developed.

We provided the final version of the scale to (*N* = 13) residents in psychiatry to check the visibility and preference, and to ensure the understanding of each item by the participants. For each word questioned by participants or not understood, the final version was reviewed and adapted by the two translators by consensus. The step of reviewing the results of the application and finalization allows us to make the last necessary changes in the preliminary tool. The instructions for the various items were well understood by clinicians and were easily administered; few reformulations were necessary (Table 9).

**Table 9:** SSTIC – (Sinhala version).

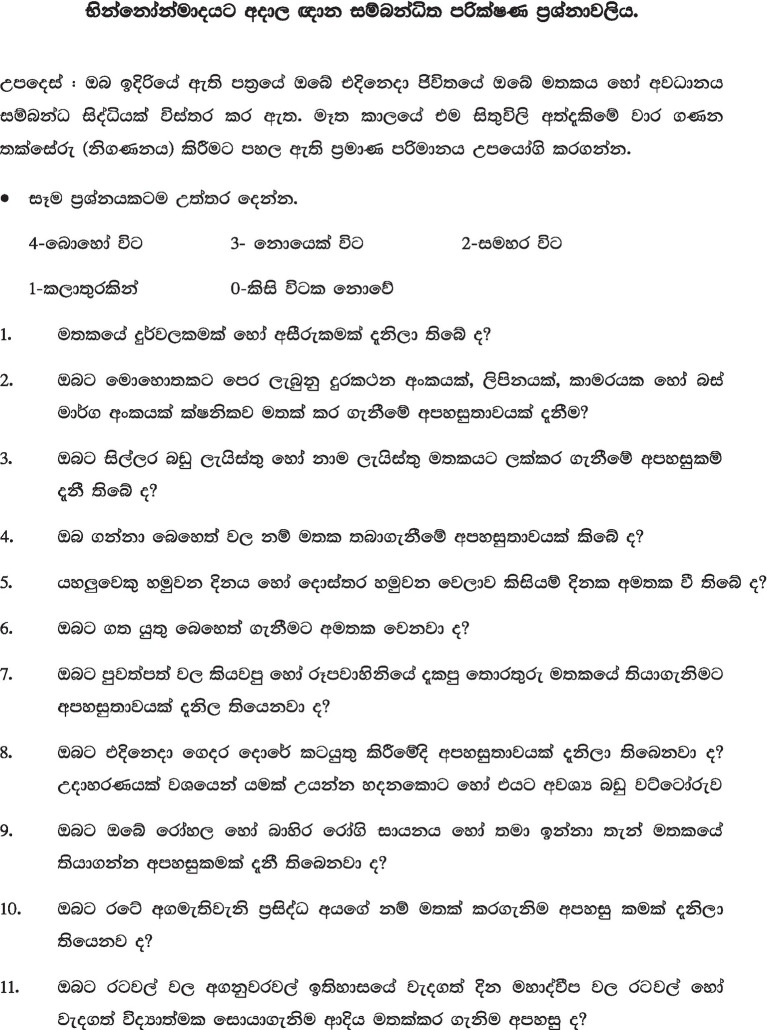
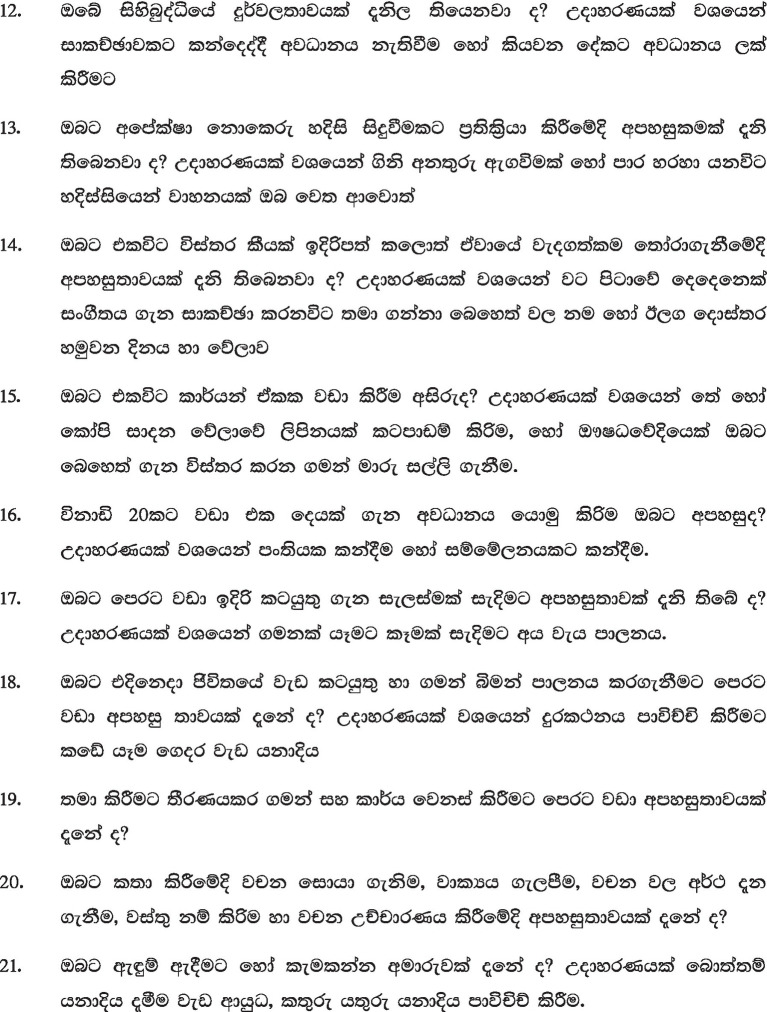

## Results

### Results (Arabic)

The Arabic language is known for its complexity and richness of vocabulary. Its singularity appears from its designation. If one mentions Arabic without an adjective, some will understand that it is classical Arabic, which is also called literary, literal, or Koranic. Others perhaps, less numerous, would consider it dialectal Arabic, i.e., one of the forms of Arabic spoken in the Maghreb like Tunisia or Saharan Africa (Mauritania) or the East (Egyptian, Sudanese, Chadians, Saudis, Jordanians, Palestinians, Lebanese, Syrians, Iraqis, speak of Dathina, Hadramawt, Yemeni, and Omani).

**Figure 2:**
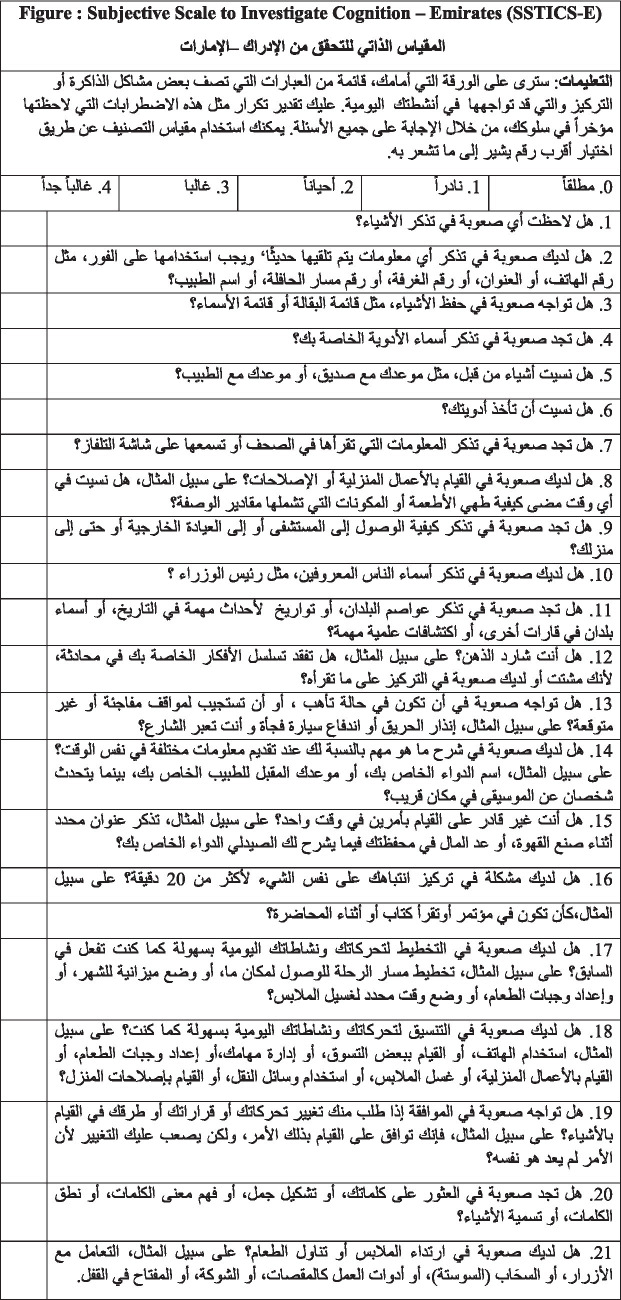
Subjective Scale to Investigate Cognition-Emirates (SSTIC-E).

Diglossia that prevails in the Arab world, namely the existence of a literary variant reserved for written use and the dialectal variants that are spoken in the various Arab countries, might be very old. The Koranic Arabic was not a spoken language whose dialects would be emitted historically but a standard of prestige in which the prophet received and wanted to transmit the revelation of Islam. In addition, literary Arabic is currently used in all Arabic literature. It can be used on the radio and television, in official speeches, in scientific communication, and in any other formal circumstance (Figure 2).

What nevertheless makes it possible to say that literary Arabic is not a spoken language in the ordinary sense of the term is, in fact, simple: it is not the mother tongue of people; the inhabitants of Arab countries speak and transmit to their children only the dialect of the country where they live. We know that Arabic dialects can be quite different, even within the same geographic and cultural settings, and more so between two sets: an Emirati and a Moroccan may have trouble communicating, each speaking his/her dialect. In this case, using literary Arabic may be considered. However, because of the substantial heterogeneity of the levels of schooling and education, not every Arabic speaker knows literary Arabic well enough to use it in their oral communication. In addition, the use of literary Arabic in oral communication appears to most Arabic speakers as being artificial, the dialects alone having a reality as instruments of conversation (Table 10).

**Table 10:** SSTICS – (Russian version).

Субъективная шкала для исследования сознания при шизофрении	
Инструкция: Вы видите на листе бумаги перед собой список фраз, описывающих проблемы с памятью или концентрацией, с которыми каждый из Вас мог сталкиваться в обыденной жизни. Просим Вас оценить частоту, с которой такие проблемы отмечаются в Вашем поведении путем ответов на поставленные вопросы. Используйте шкалу для оценки частоты путем выбора числа наиболее близкого к тому, что Вы ощущаете.	
4- очень часто − 3- часто − 2- иногда − 1- редко − 0- никогда	
1- Заметили ли Вы какие-либо сложности в запоминании вещей?	
2- Испытываете ли Вы сложности в запоминании информации, которая только что к Вам поступила и должна быть использована немедленно, например, номер телефона, адрес, номер комнаты, рейсовый номер автобуса или имя доктора?	
3- Испытываете ли Вы сложности запоминания таких вещей как список продуктов или список имен?	
4- Сложно ли Вам держать в памяти наименование принимаемых Вами лекарств?	
5- Вы когда-либо забываете о таких вещах, как встреча с другом или запись на прием к доктору?	
6- Забываете ли Вы принимать лекарства?	
7- Испытываете ли Вы сложности запоминания информации, которую прочли в газетах или услышали по телевидению?	
8- Испытываете ли Вы сложности при выполнении работ по дому или ремонта? Например, забывали ли Вы когда-либо, как готовить пищу или какие ингредиенты входят в рецепт приготовления?	
9- Испытываете ли Вы сложности в запоминании, как пройти в больницу или поликлинику или даже вернуться к себе?	
10- Сложно ли Вам запомнить имена известных личностей, например, Премьер министра Вашей страны?	
11- Испытываете ли Вы сложности запоминания столиц государств, важных дат в их истории, наименовании стран на других континентах, или основных научных открытий?	
12- Вы рассеянны или витаете в облаках? Например, вы теряете ход мыслей в разговоре, потому что отвлекаетесь или вам трудно сосредоточиться на том, что вы читаете?	
13- Вам сложно быть начеку или реагировать на неожиданные ситуации? Например, на пожарную сигнализацию или машину, которая внезапно проезжает мимо, когда вы переходите улицу.	
14- Сложно ли вам отличить, что важно, когда вам одновременно подается разная информации? Например, название Вашего лекарства или следующий визит к врачу, когда рядом два человека говорят о музыке.	
15- Вы не можете выполнять две задачи одновременно? Например, запоминать адрес во время приготовления кофе или подсчитывать деньги в кошельке, пока фармацевт объясняет Вам о лекарстве.	
16- Вам сложно сосредоточить внимание на одном и том же более 20 минут? Например, на конференции или читаемой книге или уроке в классе.	
17- Ощущаете ли Вы, что Вам уже не настолько легко планировать свою деятельность, как раньше? Например, составление графика поездки, составление бюджета на месяц, приготовление еды или отведение времени на стирку.	
18- Ощущаете ли Вы, что Вам сложнее стало координировать свои движения и действия в повседневной жизни, чем это было раньше? Например, использование телефона, покупка, выполнение поручений, приготовление еды, работа по дому, стирка, пользование услугами транспорта, проведение домашнего ремонта.	
19- Сложно ли Вам изменить Ваши движения, решения или способы действия, если Вас просят об этом, и вы согласны? Например, вы согласны поступить так, но это составляет сложность, потому что это уже не то, что раньше.	
20- Трудно ли Вам находить слова, составлять предложения, понимать значение слов, произносить слова или называть предметы?	
21- Вам трудно одеваться или кушать? Например, застегивать пуговицы, молнии, управлять рабочими инструментами, ножницами, вилкой, ключом от замка.	

### Translating and back-translating: from SSTICS to SSTICE

The initial translation aims to develop the first version of the tool in the language used in the UAE. A native speaker in both languages Arabic and English performed the initial translation. The process of adopting the SSTICS to produce the SSTIC-E was performed through four main stages:

- The first stage was the examination of the two versions of SSTICS, the initial French language version that was translated to Tunisian Arabic (SACCS) and the English language version (SSTICS).
- The second stage was the translation (by a single researcher) of the English SSTICS into the Arabic Emirati version language, named the Subjective Scale to Investigate Cognition in Emirates (SSTIC-E).
- In the third stage involved translating the two Arabic SSTICS versions (SASCCS and SSTIC-E) back into the English language. Two independent researchers processed each scale version, each blinded to the other “investigator’s work.” After completing this stage, we obtained four different back-translations (two back-translations for each scale), performed by four independent researchers.
- In the fourth stage, one reviewer compared the four translations and chose the version most like the original English-language SSTICS, so that adaptation and validation could be performed, and the final version of SSTICE-E was presented (Figure 1). The author of the scale (ES) reviewed all the versions.

Finally, in the harmonization stage, meetings were held between the different people involved in translation to examine the divergent items and concepts and ensure inter-translation validity. During these meetings, participants made decisions regarding concepts identified as potentially problematic or confusing in the meaning and understanding of the instructions. When necessary, the author of the tool has provided clarifications on the meaning of the concepts so that the Arabic version is as accurate as possible to the original. Decisions made by meeting participants were noted, and a draft of the tool was developed.

### SSTIC-E piloting

We provided the final version of the scale to 13 residents in the Al Ain Hospital psychiatry clinic, the main goal was to check the translated version in terms of wording adaptability, visibility, and preferred language (Arabic or English) of using the SSTIC-E, as well as to ensure the understanding of each item of the scale by the participants. No obstacles were documented by participants, and all their comments were taken into consideration for a better and clearer version of the final Arabic version of the scale. The final version was reviewed and adapted by the two translators by consensus.

### Additional step: comparison for SSTICS back-translation of 9 languages

To enhance the importance of linguistic and textual differences in a different type of cross-translation and cross-adaptation based on each country’s language, context, and culture, we received different versions of back-translation from different countries. All the unpublished translations are displayed in the supplementary section. These versions of the scales are *Pakistani/Urdu, Hindi, Marathi, Lithuanian, Serbian, German, Romanian, Sinhala, and Russian*.

When we compared the back-translations, we found slight differences in using alternative synonymous words without changing the general meanings of the sentences or the questions. Moreover, we found some of the back-translations had slightly different grammar, others added more words or used completely different vocabulary (for example, in some back-translations, they used “you face difficulty” and in other back-translations “you struggle,” also, some used “house maintenance” and some used “home repairs”).

Some of the back-translations we found typical as the original scale, those highlighted in light orange (original scale BT, and BT-*Version 1, 3*) and others also had typical back translations but differed slightly from the original scale, highlighted in light green (*BT-Version 4, 5*). We classified differences into two types: grammarian, and semantic and contextual.

### Grammarian

Because most of the back-translations come from countries where English is not the first language, we have noticed some mistakes in grammar and punctuation. These variations may affect the context meanings because of their misplaced positions in the sentence or using the wrong verb or noun sometimes.

### Semantic

Context and culture: The socio-political context would also have influenced the formulation of the question. For example, “Who is the first minister of Canada?” (Q #10) is a question related to explicit semantic memory. In some countries, the election of a first minister or president of the republic is every 4 or 5 years. In other countries, there is no election, and the leader is for life; this is the case with monarchical models. In the use of the scale in France, for example, we will instead ask who the president of the Republic is. In the Emirates, the question would be “What is the name of the leader of the country?”

In addition, the sociological context is not the same relative to cooking, laundry, etc. For example, Q.17 tests the executive tasks, but in the UAE culture, this question is not significant since Emirati citizens do not do laundry themselves, instead, they rely on house helpers or automatic laundries (see Supplementary Table S1). Considering that the SSTICS was developed from a construct of cognition, the adequacy between the question on executive functions and real life is crucial if one wants a minimum of ecological validity (Supplementary Table S1)

## Discussion

### Precautions

In this process, we had to choose between different methods such as the traditional translation. It simply consists of the translation by a bilingual researcher or professional translator of the original instrument. This method, used alone, is not recommended because it introduces significant bias, especially in the interpretation of the researcher or the translator. This difficulty can be overcome by performing several parallel translations by different bilingual translators or researchers, but the following methods are recommended. Therefore, the team was composed of several psychiatrists from different cultures within the Arab world.

The method of translation by a committee of experts involves participation in the translation of several bilingual people who know the field for which the instrument is intended, which also limits the bias of a single researcher. This committee can consider a first translated version or participate in the development of a first version. Under ideal conditions, if the author (ES) of the original version participates in it, this makes it possible to clarify certain ambiguities that the translation process generates. We were able at this stage to document certain differences in dialect and semantic usage. For instance:

Comments: “some minor comments on the translation.”

Some repeated words, some literal translations, and redirecting for the correct meanings of the words in Arabic. Here are some examples: all the texts written in Arabic are accompanied by an interlinear gloss, to help the reader follow the relationship between the source text and its translation, and the structure of the original language:


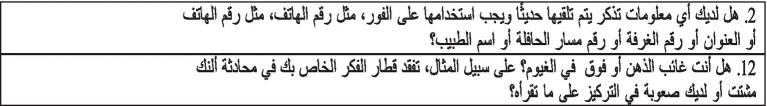


التعليمات: سترى على الورقة التي أمامك، قائمة من العبارات التي تصف بعض مشاكل الذاكرة أو التركيز والتي قد يواجهها كل منكم في أنشطته اليومية.جميع األسئلة عليك تقدير تكرار مثل هذه االضطرابات التي الحظتها مؤخراً في سلوكك، من خالل اإلجابة على.

2- Do you have difficulty remembering information that is freshly received and that must be used immediately, such as a telephone number, an address, a room number, a bus route number, or a doctor’s name?

12-Are you absent-minded or up in the clouds? For example, you lose your train of thought in a conversation because you are distracted, or you have a hard time focusing on what you are reading?

Back-translation implies that when the first version translated from the instrument has been produced, it is re-translated by a second person in its original language. The gap between the original version and the re-translated version identifies problematic items. This method can be even more sophisticated by performing two reverse translations in parallel, involving four people. This method can be considered ideal and was used. However, researchers who have used it find it challenging to obtain a perfect equivalence between the re-translated and original versions. For instance: Q# 20 Do you have difficulty finding words, making sentences, or understanding the meaning of words (may need to specify whether you mean written or spoken words)? or pronouncing them, or naming objects?

In addition, a recent study by Cella and colleagues showed that the SSTICS could be used as a screening measure and be completed by service users independently (Cella et al., 2020). However, item complexity may limit self-administration, which can affect item interpretation (Eremenco et al., 2005).

### First use in UAE: exploratory and feasibility findings

As a first opportunity, a population of our area and hospital agreed to participate in our questionnaire when they had a follow-up during the COVID-19 pandemic. This gave us the opportunity to test the use of the SSTIC-E (UAE Arabic) with a sample of 23 subjects. The mean age of our sample was 30.18% (18–62) 62% were female. The mean SSTICS total score was 16.5 (SD:16.9), in accordance with scores found in previous studies using the SSTICS in similar samples (Mancini et al., 2002). Working memory 2.13 (SD 1.83); Explicit memory 6.20 (SD 6.02); Attention 4.65 (SD 4.12); Language 1.13 (SD 1.06); Praxis 0.19 (SD 0.46). No correlations were found with age since this sample size was small. The Ethical Board at UAEU accepted the project and every subject gave consent to participate.

Second, and although this article is not focused on carrying out a clinical validation study at this stage but rather on describing the processes to be respected beforehand in the cultural and linguistic validation of the construct, we summarize here the feasibility results obtained from a population in the Emirates: The Arabic version of the SSTIC-E was administered to a total of 210 participants, including 126 patients and 84 healthy control participants (Al Mugaddam, 2023). The healthy group has a lower mean score of 22.55 (SD = 12.04), with a range of scores from 1 to 63. On the other hand, the patient group has a higher mean score of 34.06 (SD = 15.19), with a range of scores from 1 to 73 (*p* < 0.001). Additionally, the scores are broken down into five cognitive domains: Memory, Attention, Executive Function, Language, and Praxia. In the Memory domain, the control group has a mean score of 13.50 (SD = 7.21), with a range of scores from 1 to 36, while the patient group has a mean score of 17.58 (SD = 8.22), with a range of scores from 0 to 38; (*p* = 0.0005). In the Attention domain, the control group has a mean score of 5.81 (SD = 3.65), with a range of scores from 0 to 16, while the patient group has a mean score of 9.68 (SD = 4.64), with a range of scores from 0 to 20; (*p* < 0.0001). In the Executive Function domain, the control group has a mean score of 3.01 (SD = 2.52), with a range of scores from 0 to 12, while the patient group has a mean score of 4.76 (SD = 3.29), with a range of scores from 0 to 12; (*p* = 0.0002). In the Language domain, the control group has a mean score of 0.90 (SD = 9.6), with a range of scores from 0 to 4, while the patient group has a mean score of 1.45 (SD = 1.25), with a range of scores from 0 to 4; (*p* = 0.0786). In the Praxia domain, the control group has a mean score of 0.24 (SD = 0.63), with a range of scores from 0 to 3, while the patient group has a mean score of 0.76 (SD = 1.15), with a range of scores from 0 to 4; (*p* = 0.0009). To explore comparison, we compared the proportions between the control and patient groups for the SSTIC-E Scores, performing independent sample t-tests for each domain of the SSTIC-E score. In all domains except for Language, the mean scores of the patient groups were significantly higher than those of the control group, indicating worse performance in cognitive functioning. The effect sizes (Cohen’s d) were moderate to large for all domains except for Language, where the effect size was small. Overall, the patient group has higher scores in all domains, indicating that they have more cognitive impairment than the control group. Comparing the study results with the literature, the total mean score on the SSTICS for Stip et al.’s (2003) study population was 25.94 (SD = ±9.72) (Stip et al., 2003), which is similar to the study findings by Raffard et al. (2020) in France, where patients scored 25.56 (SD = ±9.10), but lower than the score of our patient participants, 34.06 (SD = ±15.19). However, the control group in the latter study scored similar results (22.55, SD = ±7.87) (Raffard et al., 2020) to the control group of our study (22.55, SD = ±12.04).

Most of the studies had SSTICS mean scores relatively close to our study results. For example, Stratta et al. (2020) found a mean score of 23.34 (SD = ±14.91) when validating the SSTICS in an Italian patient population (Stratta et al., 2020), while Lecardeur et al. (2009) reported a mean score of 24.73 (SD = ±9.56) for patients with schizophrenia who had preserved awareness of their cognitive deficits, as measured by the SSTICS (Lecardeur et al., 2009). In contrast, Haddad et al. (2021) found that subjective cognitive complaints were associated with poorer cognitive performance in Lebanese patients with schizophrenia, with patients scoring 25.15 (SD = 16.67; min = 0, max = 76; median = 23.50) and controls scoring 9.15 (SD = 7.63; min = 0, max = 37; median = 7.00) (Haddad et al., 2021). The lowest mean score for SSTICS among patients was reported by Baliga et al. (2020) in India, with a mean score of 16.22 (SD = ± 10.8) (Baliga et al., 2022). Similarly, Shin et al. (2016) in Korea reported a mean SSTICS score of 18.9 (SD = ±14.3) for patients, Shin et al. (2016) and Chuang et al. (2019) in Taiwan found a mean SSTICS score of 16.22 (SD = ± 10.8) for their study population of patient participants (Chuang et al., 2019).

### Applications

The intended application of translated versions is multiple depending on the languages and countries where the scale may be useful. In the Emirates, it gives the possibility of choosing a version, depending on the origin of the person tested, their mother tongue, or the language they use at home, so that the complaint best reflects their cognitive reality. The diversity of the authors of the article reflects this concern and may reflect the motivation of a healthcare professional to use the scale with a variety of people. Additionally, the scale can be used by professionals who propose a cognitive remediation plan. For example, neurocognitive disorders, which affect approximately 80% of people with schizophrenia, are present from the first psychotic episode. They must be objectified as soon as possible. A neuropsychological evaluation is considered when a cognitive complaint is formulated. Such a complaint may initially be raised by the SSTIC-E. In this case, a neuropsychological assessment and an assessment of social cognition are implemented. In the end, the results must be returned to the patient, so that they can take ownership of them. The preserved processes on which they can rely on to recover are put into perspective with those that are altered so that they can manage them as best as possible. The application will also eventually be broadened in transdiagnostic approaches. In certain geographic or economic situations, there are no means to carry out a complete neuropsychological assessment. The scale will still provide a signal of cognitive difficulty. Finally, the scale can be used in clinical or epidemiological research.

## Conclusion

In summary, in the back-translation, the translation from the languages used in the UAE, including Arabic, was re-translated into English. Subsequently, a revision of the reverse translation was performed. This involves comparing the version translated back into English with the original version of the measurement tool to ensure the equivalence of the concepts. Both versions have been revised to identify any discrepancies. Problematic items were reviewed, and the Arabic version and other languages were improved accordingly.

Instrument developers face many challenges in developing translations. Multi-step procedures should be used, with bilingual and bicultural experts working independently or in committee and using mixed qualitative and quantitative methods to test translations. It remains difficult to achieve 100% equivalence (Beaton et al., 2000).

The study presented provides the first version of SSTICS intended for cross-cultural validation. The role of the translator’s team was to produce a text that could convey meaning to the receptors of the target culture, i.e., in the Gulf Cooperation Council countries (GCC). The team tried to respect the rule of intra-textual coherence, which stipulates that the target text (translates) must be sufficiently intelligible for the receiver and have a communicative and cultural meaning, as a part of his world of reference, for instance in Dubai or Abu Dhabi. The future use of the SSTIC-E cross-culturally adapted to the UAE will be a starting point for comparisons and further studies using this scale in the UAE and the Gulf region. Epidemiological studies using assessment scales such as SSTICS help bridge healthcare professionals and policymakers and help establish the best medical practices, policies, and interventions.

## Data availability statement

The raw data supporting the conclusions of this article will be made available by the authors, without undue reservation.

## Ethics statement

The studies involving humans were approved by United Arab Emirates University, Social Sciences Ethical Committee (UAEU-SSeC), (ERS_2021_8418). The studies were conducted in accordance with the local legislation and institutional requirements. The participants provided their written informed consent to participate in this study.

## Author contributions

ES created the scale and the design of the study, supervised the process, and wrote the first version of this manuscript. FA translated SSTICS from English to Arabic and the final modification of the Arabic scale (SSTIC-E). LA and KA made substantial contributions to the first back-translation of SSTICS from the Arabic version into English. AO made substantial contributions to the second back-translation of SACCS, the Arabic Tunisian version into English. DA made substantial contributions in comparing the four back-translations and chose the most similar one to the original SSTICS. SJ was responsible for the version in Urdu. AS and RB were responsible for the versions in Hindi and Marathi. SW was responsible for the Sinhala version. EA was responsible for the German version. VA was responsible for the Lithuanian version. AM-M was involved in the first study with SSTICS and reviewed the manuscript. All authors contributed to the manuscript and accepted to be accountable for the content of the study.
