# Supplementary material for: Cross-cultural differences through subjective cognition: illustration in translatology with the SSTIC-E in the UAE
Source: Front Psychol. 2024 Mar 7;15:1125990. doi: 10.3389/fpsyg.2024.1125990 (PMC10956416; doi:10.3389/fpsyg.2024.1125990)
Supplement: Supplementary file 1 [file Table_1.docx]

| **Scale BT- Versions** |  |  |  |  |  |  |  |  |  |  |  |
| --- | --- | --- | --- | --- | --- | --- | --- | --- | --- | --- | --- |
|  | ***BT-*** **Version 1**  *(Emirate)* | **BT-Version 2**  *(Pakistani-Urdu))* | **BT-Version 3**  *(Hindi)* | **BT-Version 4**  *(Marathi)* | **BT-Version 5**  *(Lithuanian)* | | **BT-Version 6**  **(** *Serbian)* | **BT-Version 7**  *(German)* | **BT-Version 8**  **(** *Romanian)* | **BT-Version 9**  *(Sinhala)* | **BT-Version 10**  *(Russian)* |
| **Title** | Subjective scale **to investigate** cognition | Subjective scale **to assess** cognition | Subjective scale **to investigate** cognition | SSTICS: Back Translation | SSTICS: Back Translation | | Subjective scale for assessing cognition in schizophrenia | Subjective scale for examining perception. | Subjective Scale for Cognitive Investigation. | Questionnaire related to Schizophrenia | Subjective scale for the study of consciousness in schizophrenia |
| **Scoring** | 0 never  1 rarely  **2 sometimes**  **3 often**  **4** very often | 0 never  1 rarely  **2 occasionally**  3 often  **4 very frequently** | 0 never  1 rarely  2 sometimes  3 often  4 very often | 0 never  1 rarely  2 sometimes  3 often  4 very often | 0 never  1 rarely  2 sometimes  3 often  4 very often | | 0- never  1- rarely  2- sometimes  3- often  4- very often | 0 – never  1 – rarely  2 – sometimes  3 – often  4 - very often | 0- never  1-rare  2-sometimes  3-often  4- very often | 0 – never  1 – seldom  **2 – maybe**  **3 – At various times**  4 – often | 0- never  1- rarely  2- sometimes  3- often  4- very often |
| **Q1** | Have you noticed any **difficulty** **remembering things**? | Have you noticed any **problems** with your **memory**? | Have you noticed any **difficulty remembering** things? | Have you noted any **difficulties** in **remembering things**? | Have you noted any **difficulties in remembering things**? | | Have you noticed any memory difficulties? | Do you have trouble **remembering things?** | Do you have a hard time remembering things? | Have a weak memory or **difficulty?** | Did you notice any **difficulty in** **remembering things?** |
| **Q2** | Do you have **difficulty remembering information** that is **freshly received** and **that** must be **used immediately**, such as a telephone number, an address, a room number, a bus route number or a 'doctor's name? | Do you **experience problems** **remembering new information** that has to be **used immediately**? For example, remembering a phone number, an address, a room number, a bus route or your 'doctor's name? | Do you have **difficulty remembering information** that is **freshly received** and **that** must be **used immediately**, such as a telephone number, an address, a room number, a bus route number or a 'doctor's name? | Do you **have difficulties in remembering information** you have **just received** that you need to use immediately, such as a telephone number, an address, a room number, a bus number, the name of a doctor? | Do you **have difficulties in remembering information** you have **just received** that you need to use immediately, such as a telephone number, an address, a room number, a bus number, the name of a doctor? | | Do you **have difficulty remembering newly received information** that you must use immediately, such as phone number, address, room number, bus route number, or doctor's name? | Are you **having trouble remembering information that** you just received that you need to use again immediately, such as: a telephone number, an address, a room number, the number of a bus line or the name of a doctor? | Do you **have difficulty remembering information** that you have just learned that you need to use immediately, such as a phone number, address, room number, bus number, or a doctor's name? | Feeling any **difficulty in suddenly remembering** a phone number, address, room, or bus number you received a moment ago? | Do you experience **difficulties remembering information** that has just come to you and should be used immediately, for example, phone number, address, room number, a regular bus number or a name of a doctor? |
| **Q3** | Do you **have difficulty** **memorizing** things, **such as** a **grocery list** or a list of names? | Do **you experience** **problems remembering certain** things **like shopping list** or list of names? | Do you **have difficulty memorizing** things, **such as** a **grocery list** or a list of names? | Do you **have any difficulties memorizing** things, **such as a list of food items** or names? | Do you **have any difficulties in memorizing** things, **such as a list of food items** or names? | | Do you **have difficulty** **remembering** things, like a grocery list or a name list? | Do you have **trouble memorizing** things, for example, the shopping list or a list of names? | **Having trouble remembering** a grocery list, or names? | **Have you ever had trouble remembering** groceries or name lists? | **Do you experience difficulties remembering** information that has just come to you and should be used immediately, for example, phone number, address, room number, a regular bus number or a name of a doctor? |
| **Q4** | Do you **have difficulty remembering** the **names of your medications**? | Do you **find it difficult to remember** your **medication list?** | Do you **have difficulty remembering** the **names of your medications**? | Do you **struggle to remember** the **name of your medications?** | Do you **struggle to remember** the **name of your medications**? | | Do you have difficulty **remembering** the names of the medications you are taking? | Are you having trouble **remembering** the names of your medications? | Do you have **difficulty** **remembering** the name of your medications? | Do you have **difficulty remembering** the names of the medications you are taking? | Is it hard for you to **remember the name** of the drugs that you take? |
| **Q5** | **Do you ever** **forget things**, **such as a date** with a **friend or a 'doctor's appointment**? | **Do you forget to attend meetings** with **your friends or appointments arranged with your doctor**? | **Do you ever forget thing**s, **such as a date with a friend or a 'doctor's appointment?** | **Do you ever forget things**, such as **a meeting with a friend or an appointment with the doctor?** | **Do you forget things,** such as **a meeting with a friend** or **an appointment with the docto**r? | | Do you ever **forget things, like a meeting** with a friend or an **appointment** with a doctor? | **Do you forget things** like a **date with a friend or a doctor's appointment?** | Do you sometimes **miss an** **appointment** with a friend, or at your doctor? | Have you ever **forgotten the date of** seeing a friend or the time of seeing the doctor? | Do you ever f**orget about such things as meeting** a friend or an appointment with a doctor? |
| **Q6** | **Do you forget** to **take your medication?** | **Do you forget** t**aking** your **medication**? | **Do you forget to take** your **medication**? | **Do you forget to take** your **medications?** | Do you **forget to take your medications?** | | Do you **forget to take medication?** | Do you **forget to take your medication?** | Did you **forget to take your medication** occasionally? | Do you forget to **take the medicine** you need to take? | Do you **forget to take your medications**? |
| **Q7** | Do you **have difficulty remembering** information that you read in the newspapers or hear on  TV? | Do you **struggle to recall information** that you read in the newspapers or hear on Television? | Do you **have difficulty remembering** information that you read in the newspapers or hear on TV? | Do you **struggle to remember** information read in the newspapers or heard on TV? | Do you **struggle to remember** information read in the newspapers or heard on  TV? | | Do you have **difficulty remembering** information you read in the newspaper or hear on TV? | Do you have **trouble remembering** information you read in the newspaper or heard on TV? | Do you have trouble **remembering new readings** in the newspapers or on TV? | Have you ever **had difficulty remembering** what you read in the newspapers or saw on television? | Do you have difficulty **memorizing information** that you read in newspapers or heard on television? |
| **Q8** | Do you **have difficulty doing household** **chores** **or repairs?** For example, do you ever **forget how to cook things** or what **ingredients go into a recipe**? | Do you **experience problems in completing household** **chores and repairs?** For example, do you **tend to forget** how to **prepare meals** or what **recipe ingredients to use**? | Do you have **difficulty doing household chores or repairs**? For example, do you ever **forget how to cook things** or what **ingredients go into a recipe**? | Do you have **difficulty doing household chores or repairs?** For example, do you ever **forget how to cook things** or what **ingredients go into a recipe?** | Do you have **difficulties to carry out household chores or maintenance duties**? For example, **do you forget to** **prepare food** or the ingredients of the recipe? | | Do you have **difficulty doing household** chores or repairs? For example, do you ever forget how to cook a dish or what ingredients are in the recipe? | Are you having **difficulty** **doing household** chores or repairs? For example, do you forget how certain things are cooked or which ingredients belong to a certain recipe? | Do you have **difficulty doing work or repairs in** your home? For example, have you forgotten how to prepare, or what ingredients you need for a recipe? | Have you ever **felt uncomfortable doing daily housework?** For example, when trying to cook something or the ingredients needed for it | Do you have **difficulty doing household duties or repairing tasks?** For example, have you ever forgot how to prepare food or what ingredients are included in its recipe? |
| **Q9** | Do you have **difficulty remembering** how **to get to the hospital** or the **outpatient clinic** or even to **your own place**? | Do you **have problems finding** your way to hospital, **clinic** or even to **your own home**? | Do you **have difficulty remembering** how **to get to** **the hospital** or the **outpatient clinic** or **even to your own place**? | Do **you struggle to remember** how to reach the hospital, the outpatients of **your place of residence**? | Do you **struggle to remember** how to reach the hospital, the outpatients of **your place of residence**? | | Do you have **difficulty remembering** how to get to a hospital or clinic or even to your home? | Do you have **trouble remembering** how to get to the hospital or ambulance or home? | Do you have **difficulty trying to get to** the hospital, the polyclinic, or even your home? | Have you ever had **difficulty remembering** your hospital or outpatient clinic or where you are? | Do you have **difficulty remembering** how to get to a hospital or polyclinic, or even return back to your place? |
| **Q10** | **Do you have difficulty** remembering the names of **well-known people**, such as the **Prime Minister** of **Canada?** | **Do you have difficulty remembering** the names of **famous personalities**? For example, who is the **current prime minister** of **Pakistan?** | Do you **have difficulty** remembering the names of well-known people, such as **the leader of the country**? | Do you **struggle with** remembering the name of **famous individuals**, such **as the president of the Republic**? | | Do you **struggle with** remembering the name of **famous individuals,** such **as the president of the Republic?** | Do you have **difficulty remembering** the names of famous people, such as the President of Serbia? | Do you have **trouble remembering** the names of people you know? **the name of a leader?** | Do you have **difficulty remembering** the names of well-known people, such as **the president of your country?** | Do you find it **difficult to remember** the names of famous people like **the Prime Minister of the country?** | Is it difficult for you to **remember names of** famous personalities, e.g., **the Prime Minister of your country?** |
| **Q11** | **Do you have difficulty** **remembering national** **capitals,** important dates in history, **names of countries on other continents**, **or major scientific discoveries?** | **Do you have trouble remembering capital cities**, important historic events, countries, continents **or major scientific discoveries?** | **Do you have difficulty remembering national** **capitals**, important dates in history, **names of countries on other continents**, **or major scientific discoveries?** | **Do you struggle to remember names of countries capitals cities**, important **historical events**, **names of countries** **in other continents, or major scientific discoveries**? | | **Do you struggle to remember names** of countries capitals cities, important historical events, names **of countries in other continents, or major scientific discoveries**? | Do you **have difficulty remembering** national capitals, important dates in history, names of countries on other **continents or great scientific discoveries**? | Do **you have trouble remembering** state capitals, important dates in history, names of countries on other **continents, or important scientific discoveries?** | Do you have difficulty remembering the names of country capitals, important dates in history, or the names of countries on other **continents, or major scientific discoveries?** | Do you find it **difficult to remember** the important days in history, capitals of the countries, the countries of the **continents or the important scientific discoveries?** | Do you experience **difficulties in remembering** capitals of states, important dates in their history, the names of countries on other **continents, or major scientific discoveries?** |
| **Q12** | **Are you absent-minded or** **up in the clouds**? For example, you **lose your train of thought in a conversation** because you are **distracted or you have a hard time focusing on what you are reading**? | **Do you find yourself puzzled or mixed up on occasions**? For example, **you cannot focus on conversations when disturbed or distracted** or **struggle to concentrate on tasks like reading**? | **Are you absent-minded or up in the clouds**? For example, you l**ose your train of thought in a conversation** because you are **distracted or you have a hard time focusing on what you are reading?** | **Are you easily distractible or lack attention**? For example, do you **lose flow of words during a conversation** because you lack attention during a conversation or **struggle to focus on what you are reading**? | | **Are you easily distractible or lack attention?** For example, do you **lose flow of words during a conversation** because you lack attention during a conversation or **struggle to focus on what you are reading**? | **Are you absent or lost in thought?** For example, do you **lose the flow of thoughts during a conversation** because you are distracted or do you find **it difficult to focus on what you are reading?** | Are you inattentive or **absent-minded?** For example, do you **lose the thread of a conversation** because you are distracted or find **it difficult to focus on what you are reading?** | Do you feel lost, or your head in the clouds? Like for example you **lose the thread of your thoughts in a conversation** because you are distracted, or you find **it difficult to focus on your reading?** | Have you ever felt weak in your mind? For example, **lose focus while listening** to a discussion or when **focusing on what is being read?** | Are you absent-minded or in the clouds? For example, do you **lose your thoughts in a conversation** because of being distracted or do you find it difficult to focus **on what you read?** |
| **Q13** | **Do you have difficulty being on the alert or reacting to unexpected situations?** For example, a **fire alarm or a car that rushes by suddenly** as you are **crossing the** **street**. | **Do you struggle to keep your attention levels up when faced with unexpected circumstances?** Like when **you hear the fire alarm go off or a car whizzes past** you as you are about **to cross the road**. | **Do you have difficulty being** on the alert or **reacting to unexpected situations?** For example, a **fire alarm or a car that rushes by suddenly as you are crossing the street**. | **Do you struggle to keep** **your attention** **levels up** **when faced with** **unexpected circumstances**? Like when you hear **the fire alarm go off or a car whizzes past** you as you are about to **cross the road.** | | **Do you struggle to remain** alert in the context **on unexpected situations**? For example, **a fire brigade** or **a car appearing** suddenly whilst you are **crossing the street**? | Do you **have difficulty** being alert or reacting to unexpected situations? For example, a fire alarm or a car rushing by as you **cross the street.** | Do you **have trouble** being alert or reacting to unexpected situations?  For example, a fire alarm or a car that suddenly sped by when you **cross the street.** | Do you **have difficulty** staying alert, staying focused, or reacting to unexpected situations? Like for example a fire alarm, or a car that surprises you when you cross the street? | Have **you ever felt uncomfortable** reacting to an unexpected emergency situation? For example, if a fire alarm goes off or a vehicle suddenly comes to you while **crossing the road** | **Is it difficult** for you to be alert or react to unexpected situations? For example, a fire alarm or a car that suddenly passes by when you **cross the street.** |
| **Q14** | Do you have **difficulty making out 'what's important when you are presented with different bits of information simultaneously**? For example, the **name of your medication or** your next 'doctor's **appointment while two people are talking about music nearby**? | When you are given **multiple pieces of information simultaneously**, do you find it difficult **to decide about the information more relevant to you**? For example, **remembering the name of your medication** or **the time of appointment when distracted** by some **people close by, talking about music?** | Do you have **difficulty making out 'what's important when you are presented with different bits of information simultaneously?** For example, the **name of your medication** or your next **'doctor's appointment** **while two people** are **talking about music nearby**? | Do **you struggle to prioritize** when you are presented with **several pieces of information at the same time?** For example, the **name of your medication** or your next appointment with the doctor, **when two people talk about music in close proximity**? | | Do **you struggle to prioritize** when you are presented with several **pieces of information at the same time**? For example, the **name of your medication** or your next appointment with the doctor, **when two people talk about music in close proximity**? | Do you **have difficulty discerning** what is important when you are shown different information at the same time? For example, the name of a medicine or the next check-up with a doctor while **two people are talking about music nearby at the same time.** | Are you **having trouble deciding** which information is important when you are receiving different information at the same time? For example, if you are told the name of your medication or your next doctor's **appointment while two people nearby are talking over music.** | Do you **find it difficult to discern the essentials,** when you are given information at the same time? simultaneously? Like for example the list of your medications, or the **date of the next visit to your doctor,** at the same time as other people talking about music next door? | Do you **find it difficult to choose** the importance when many details are presented to you at once? For example, when two people in the background are discussing about music, the name of the medication you are taking or the date and time to see the doctor next. | Is **it difficult for you to distinguish** what is more important when different information is presented to you at the same time? For example, the name of **your drug or your next appointmen**t with a doctor when two people discuss music nearby. |
| **Q15** | **Are you unable to do** two **things** **at once?** For example, **memorize an address while making** **coffee, or count the money** in your wallet **while the** **pharmacist explains** your medication to you. | **Can you perform** two tasks **simultaneously?** Like **recalling an address while making coffee or counting money** at the pharmacy **while listening to the pharmacist explaining your** prescription to you. | **Are you unable to do** two **things at once?** For example**, memorize an address while making coffee**, or **count the money in** your wallet **while the** **pharmacist explains your** medication to you**.** | **Are you able to carry out** two **tasks at the same time** For example, **memorize an address** **whilst you are making some coffee**, or **counting money in** your wallet **whilst the pharmacist show** you the medication**.** | | **Are you able to carry out** two **tasks at the same time** For example, **memorize an address** **whilst you are making some coffee**, or **counting money in your wallet whilst the pharmacist show** you the medication**.** | **Are you able to do two things at once**? For example, **remember the address** while making coffee or count the money in your wallet while the pharmacist explains the medication. | Are **you unable to do two things at once?** For example, memorizing an address while you prepare coffee or counting the money in your wallet while the pharmacist explains how to take your medication. | Are **you unable to do two things at the same time**? Like for example remembering an address while making coffee, or counting money from your wallet while your pharmacist tells you how to take your medications? | Do you **find it difficult to do more than one task at a time**? For example, memorizing an address while making tea or coffee, or taking change money when a pharmacist is explaining the medication to you? | Is it **difficult for you to focus attention** on one and the same thing for more than 20 minutes? For example, at a conference or a read or a lesson in class. |
| **Q16** | **Do you have trouble focusing your attention on the same thing** for more **than** 20 minutes? For example, **at a conference or a book reading or during a lesson in a classroom.** | **Can you sustain your focus on something** for **more than** 20 minutes? For example, **at a conference, while reading a book or in classroom setting.** | **Do you have trouble focusing your attention on the same thing** for **more than** 20 minutes? For example, **at a conference or a book reading or during a lesson in a classroom.** | **Do you struggle to focus** **on the same task** for **longer** than 20 minutes? For example, **during a presentation, or whilst reading a book or during a classroom lecture.** | | **Do you struggle to focus** **on the same task** for **longer** than 20 minutes? For example, **during a presentation, or whilst reading a book or during a classroom lecture.** | Do you have trouble focusing on the same thing for more than 20 minutes? For example, during a conference or while reading books or during a classroom lesson | Do you find it difficult to focus your attention on one thing for more than 20 minutes?  For example, at a conference or a book reading or during a lesson in the classroom. | Do you have difficulty concentrating on the same thing for more than twenty minutes? For example, has a lecture, a reading, or an hour in class? | Do you find it difficult to focus on one thing for more than 20 minutes? For example, attending a class or attending a conference | Do you feel that now it is not as easy for you to plan your activities as you planned before? For example, scheduling a trip, making a budget for a month, preparing meals, or arranging time for the laundry. |
| **Q17** | Do you **have difficulty planning your activities as easily as you used to**? For example, **charting an itinerary for getting someplace**, **making a budget for the month, preparing meals, or making time for laundry.** | When **compared to the past, do you find it more difficult to plan your activities now?** Like **planning to travel, budgeting, planning to prepare a meal or do the washing.** | Do you have **difficulty planning out your activities as easily as you used to?** For example, **charting an itinerary for getting someplace, making a budget for the month, preparing meals, or making time for laundry**. | Do you **struggle more than usual to plan your activities?** For example, chose an itinerary **to reach a location, budget monthly expenses, prepare food, wait for the laundry**. | | Do you **struggle more than usual to plan your activities?** For example, chose an itinerary **to reach a location, budget monthly expenses, prepare food, wait for the laundry** | Do you have difficulty planning your activities as easily as you used to? For example, to make a road plan to get somewhere, to make a budget for the next month, to prepare a meal or take time to do laundry. | Is it not as easy for you to plan your activities as it used to be? For example, planning a route to get to somewhere, setting a budget for the month, preparing meals, or taking time to do laundry. | Having trouble planning your day's activity, as you usually do? Such as setting the itinerary for a trip, or budgeting for the month, preparing a meal, or planning the next laundry | Do you find it more difficult to plan ahead than before? For example, control over who prepares food to go on a trip | Do you feel that now it is not as easy for you to plan your activities as you planned before? For example, scheduling a trip, making a budget for a month, preparing meals, or arranging time for the laundry. |
| Q18 | Do you have difficulty coordinating your movements and actions of everyday life as easily as you used to? For example, using the telephone, doing some shopping, running errands, preparing meals, doing housework, doing laundry, using transportation, doing home repairs. | When compared to the past, are still able to perform your day to day chores in an organized and structured way? Like using your phone, shopping, completing outside chores, cooking, household work, doing laundry, using transport and doing basic house maintenance. | Do you have difficulty coordinating your movements and actions of everyday life as easily as you used to? For example, using the telephone, doing some shopping, running errands, preparing meals, doing housework, doing laundry, using transportation, doing home repairs. | Do you have more difficulties than usual in coordinating day to day actions and activities? For example, using the telephone, shopping, purchasing items, preparing food, household chores, laundry, traveling by public transport, carry out repairs. | | Do you have more difficulties than usual in coordinating day to day actions and activities? For example, using the telephone, shopping, purchasing items, preparing food, household chores, laundry, traveling by public transport, carry out repairs. | Do you have difficulty coordinating your daily life and daily activities as you used to? For example, using the phone, shopping, doing business, preparing meals, housework, laundry, transportation, home repairs. | Is it no longer as easy for you to coordinate your movements and actions in everyday life as it used to be?  For example, using the phone, going shopping, running errands, preparing meals, doing household chores, washing clothes, using means of transport, making repairs at home. | Do you have difficulty coordinating your movements and actions in your daily life as you usually do? Like for example using the phone, shopping and commissions, preparing food, doing things at home, making repairs, doing laundry, or taking public transportation? | Do you find it more difficult to control your daily activities and travels than before? For example, going to the store, to use the phone or work at home | Do you feel that it became more difficult for you to coordinate your movements and actions in everyday life than it was before? For example, using the phone, shopping, running errands, preparing meals, performing household tasks, doing laundry, using transportation, or doing home repairs. |
| Q19 | Do you have difficulty changing your movements, decisions or ways of doing things if you are asked to do so and you agree? For example, you agree to do so but it is hard because it is no longer the same. | Do you have find it difficult to make changes to your ways of working, decisions and movements? For example, even when you agree with proposed changes, it is a struggle to implement the changes because of the need to use a new method? | Do you have difficulty changing your movements, decisions or ways of doing things if you are asked to do so and you agree? For example, you agree to do so but it is hard because it is no longer the same. | Do you struggle to change movements, decisions or the way you do things if you are asked if you agree? For example, you agree but then changed your mind? | | Do you struggle to change movements, decisions or the way you do things if you are asked if you agree? For example, you agree but then changed your mind? | Do you have difficulty changing movements, decisions, or the way you do things if you are asked to do so and you agree to that request? For example, you agree to do that, but it is difficult because it is no longer the same. | Do you have difficulty changing your movements, decisions, or the way you do certain things when prompted and consented to?  For example, you agree to do something, but it's hard because it's not the same anymore. | Do you have difficulty changing your movements, decisions, or way of doing things when asked and you agree? For example, you agree, but you find it difficult because it is another way of doing things, isn't it the same? | Do you have difficulty to change any pre panned travels or work? | Do you find it difficult to change your movements, decisions or methods of action if you are asked to do so and you agree to this? For example, you agree to do the change, but it is difficult because it is no longer what it used to be. |
| Q20 | Do you have difficulty finding your words, forming sentences, understanding the meaning of words, pronouncing words, or naming objects? | Do you have problems finding the right words, making meaningful sentences, understanding spoken words, pronunciation or naming objects? | Do you have difficulty finding your words, forming sentences, understanding the meaning of words, pronouncing words, or naming objects? | Do you experience difficulties in word finding, composing sentences, understanding the meaning of words, pronouncing words, or naming objects? | | Do you experience difficulties in word finding, composing sentences, understanding the meaning of words, pronouncing words, or naming objects? | Do you have difficulty finding words, forming sentences, understanding the meaning of words, pronouncing words or naming objects? | Do you have difficulty remembering certain words, having difficulty formulating sentences, understanding the meaning of words, pronouncing words or naming objects? | Do you have difficulty finding your words to formulate sentences, to understand the meaning of words, their pronunciation or to name objects? | Do you have difficulty finding words, matching sentences, knowing the meanings of words, naming objects and pronouncing words when speaking? | Do you find it difficult to find words, make sentences, understand the meaning of words, pronounce words, or name objects? |
| Q21 | Do you have difficulty getting dressed or eating? For example, handling buttons, zippers, work tools, scissors, a fork, a key in a lock. | Do you experience problems with tasks like getting dressed or eating? For example, buttoning your shirt up, using a zipper, managing tool like a pair of scissors, forks or lock and key. | Do you have difficulty getting dressed or eating? For example, handling buttons, zippers, work tools, scissors, a fork, a key in a lock. | Do you struggle in dressing or eating? For example, to bottom up clothes, lift the zipper, handle tools, scissors, a fork, or turn a key? | | Do you struggle in dressing or eating? For example, to bottom up clothes, lift the zipper, handle tools, scissors, a fork, or turn a key? | Do you have difficulty dressing or eating? For example, in the handling of buttons, zippers, work tools, scissors, a fork, or a key in a lock? | Do you have trouble getting dressed or eating? For example when handling buttons, zippers, tools, scissors, with a fork, a key in a lock. | Do you find it difficult to dress, or eat? or for example when handling buttons, or zippers, work tools, scissors, forks, or inserting the key into the door? | Do you find it difficult to dress or eat? An example is the use of buttons, tools, scissors, and so on? | Is it difficult for you to dress up or eat? For example, to fasten buttons, zippers, to operate working tools, scissors, a fork, a key in a locker. |

**SSTIC Back-Translation Versions Languages:** *BT1: Arabic Emirati; BT2: Urdu-Pakistan; BT3:* *Hindi; BT4:* *Marathi; BT5:* *Lithuanian; BT6: Serbian; BT7: German; BT8: Romanian; BT9: Sinhala; BT10: Russian*
